# DMsan: A Multi-Criteria Decision Analysis Framework and Package to Characterize Contextualized Sustainability of Sanitation and Resource Recovery Technologies

**DOI:** 10.1021/acsenvironau.2c00067

**Published:** 2023-03-27

**Authors:** Hannah A. C. Lohman, Victoria L. Morgan, Yalin Li, Xinyi Zhang, Lewis S. Rowles, Sherri M. Cook, Jeremy S. Guest

**Affiliations:** † Department of Civil and Environmental Engineering, 3221 Newmark Civil Engineering Laboratory, University of Illinois Urbana-Champaign, 205 N. Mathews Avenue, Urbana, Illinois 61801, United States; ‡ Institute for Sustainability, Energy, and Environment, University of Illinois Urbana-Champaign, 1101 W. Peabody Drive, Urbana, Illinois 61801, United States; § DOE Center for Advanced Bioenergy and Bioproducts Innovation, University of Illinois Urbana-Champaign, 1206 W. Gregory Drive, Urbana, Illinois 61801, United States; ∥ Department of Civil, Environmental, and Architectural Engineering, University of Colorado Boulder, 1111 Engineering Drive, Boulder, Colorado 80309, United States

**Keywords:** stakeholder engagement, sustainability assessment, location-specific, spatial analysis, multi-criteria decision making (MCDM), non-sewered sanitation, source-separation

## Abstract

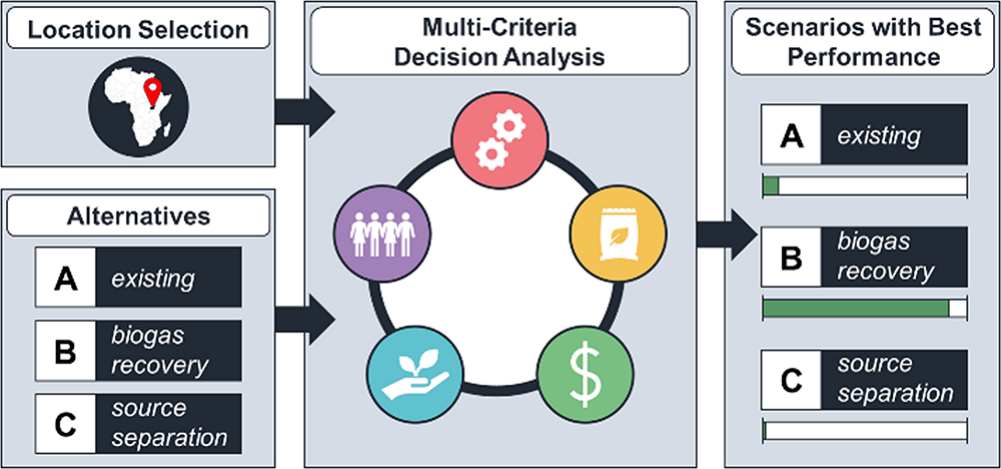

In resource-limited settings, conventional sanitation systems often fail to meet their goals—with system failures stemming from a mismatch among community needs, constraints, and deployed technologies. Although decision-making tools exist to help assess the appropriateness of conventional sanitation systems in a specific context, there is a lack of a holistic decision-making framework to guide sanitation research, development, and deployment (RD&D) of technologies. In this study, we introduce DMsan—an open-source multi-criteria decision analysis Python package that enables users to transparently compare sanitation and resource recovery alternatives and characterize the opportunity space for early-stage technologies. Informed by the methodological choices frequently used in literature, the core structure of DMsan includes five criteria (technical, resource recovery, economic, environmental, and social), 28 indicators, criteria weight scenarios, and indicator weight scenarios tailored to 250 countries/territories, all of which can be adapted by end-users. DMsan integrates with the open-source Python package QSDsan (quantitative sustainable design for sanitation and resource recovery systems) for system design and simulation to calculate quantitative economic (via techno-economic analysis), environmental (via life cycle assessment), and resource recovery indicators under uncertainty. Here, we illustrate the core capabilities of DMsan using an existing, conventional sanitation system and two proposed alternative systems for Bwaise, an informal settlement in Kampala, Uganda. The two example use cases are (i) use by implementation decision makers to enhance decision-making transparency and understand the robustness of sanitation choices given uncertain and/or varying stakeholder input and technology ability and (ii) use by technology developers seeking to identify and expand the opportunity space for their technologies. Through these examples, we demonstrate the utility of DMsan to evaluate sanitation and resource recovery systems tailored to individual contexts and increase transparency in technology evaluations, RD&D prioritization, and context-specific decision making.

## 1 Introduction

In a global push to achieve access to adequate and equitable sanitation and hygiene for all by 2030 (Sustainable Development Goal 6.2), we are only at one-fourth the necessary rate of progress to achieve this goal.^1^ Over 50% of the global population lives without safely managed sanitation systems, and 25% lives without basic sanitation.^1^ Despite efforts to improve sanitation, resource-limited settings experience failure rates up to 70% within the first 2 years of installing sanitation technologies.^2^ Even with adequate planning and funding, failures arise from critical factors not assessed during technology evaluation, such as stakeholder preferences,^3,4^ climate change and population growth,^5,6^ system operational needs,^7,8^ and user understanding and support of the system.^9,10^ Successful long-term adoption of sanitation technologies is influenced by multiple factors related to technological, environmental, economic, and social dimensions of sustainability.^11^

Decision makers, such as engineers or community planners, commonly use a suite of techniques to assess the sustainability of sanitation and resource recovery systems, including life cycle assessment (LCA) to assess environmental impacts^12−14^ and techno-economic analysis (TEA) to estimate system costs.^15,16^ However, these techniques evaluate technologies in one pillar of sustainability at a time (i.e., environment or economic) and often fail to include the social dimensions of decision making that can be a core driver of successful long-term adoption.^8^ Multi-criteria decision analysis (MCDA) supports the assessment of tradeoffs among conflicting criteria across dimensions of sustainability for decision making.^17,18^ While MCDA can be a robust approach, the criteria and indicators currently used to assess alternatives in the sanitation and resource recovery field are ambiguous and at times have conflicting definitions.^8,9^ For instance, Davis et a^8^. evaluated six decision-making frameworks^13,19−23^ intended for resource-limited settings that integrate an extensive list of 111 indicators, but reliability and social acceptability were the only technology indicators shared among all six frameworks.^8^ Furthermore, the authors evaluated 12 sanitation systems for a specific location in each framework and obtained highly varied results, where one framework ranked an option best (1st out of 12) and the other framework ranked it as second to last (11th out of 12). These studies demonstrate that MCDA can be an informative yet subjective approach, the latter of which undermines its utility when for decision makers interested in generalizable insight.

In addition to informing the locality-specific evaluation of alternatives with clearly defined stakeholder-informed criteria and indicator weights, this approach can also be used when the decision-making process, implementation context, and stakeholder priorities are still uncertain. In particular, developers of novel decentralized sanitation and resource recovery systems can benefit from identifying and expanding the opportunity space for their new technology—the contexts in which it outcompetes alternative technologies.^24^ Existing MCDA evaluation tools (e.g., TechSelect 1.0^13^ and Technology Applicability Framework^19^) are limited in their ability to evaluate the opportunity space of new technologies as a result of the (ir)relevance of weighting scenarios included (arbitrary or literature-informed weights vs context-specific stakeholder-informed weights) and the lack of consideration for uncertainty in evaluating indicators. Stakeholder engagement during early-stage technology development can help inform implementation contexts; however, limitations in time and financial resources often lead to technology evaluation with a limited number of weighting scenarios based on researcher judgment or literature.^25−27^ Additionally, existing MCDA evaluation tools often expect fixed inputs for quantitative indicators with limited flexibility for incorporating uncertainty of inputs.^28^ Fixed inputs can be problematic for early-stage technologies with high levels of uncertainty (e.g., ranges of possible cost per capita and environmental impacts),^29^ and technology evaluation with fixed inputs may not capture the entire opportunity space for new technologies.

The objectives of this work are (i) to synthesize a MCDA framework with well-defined, comprehensive indicators and context-specific weighting scenarios, (ii) to develop an open-source MCDA Python package (DMsan) that enables users (e.g., implementation decision makers and technology developers) to use this tool to transparently compare alternatives and characterize the opportunity space for technologies, and (iii) to illustrate the use of DMsan through decision making among three sanitation design configurations in a specific context. Articles centered on sanitation decision making and the assessment of two or more sanitation systems were reviewed to develop a MCDA framework with criteria and comprehensive indicators (that evaluate those criteria) commonly used in sanitation and resource recovery studies. The MCDA framework was used to develop the core structure of DMsan, which includes five criteria (technical, resource recovery, economic, environmental, and social), 28 indicators, criteria weight scenarios, and indicator weight scenarios tailored to 250 countries/territories, informed by contextual drivers. DMsan integrates with the open-source Python package QSDsan^29,30^ (quantitative sustainable design for sanitation and resource recovery systems) for the quantification of resource recovery, economic (via TEA), and environmental (via LCA) indicators under uncertainty. The DMsan package can be modified to include or exclude features as needed for an analysis, enabling users to evaluate their technologies in any country/territory and with every criteria weight combination under uncertainty. Users can characterize the opportunity space for new technologies while also charting pathways to increase sustainability, the likelihood of the technology being selected, and how best to expand the contexts in which it could be appropriate. The core capabilities of DMsan are illustrated using an existing sanitation system and two proposed alternative systems for Bwaise, an informal settlement in Kampala, Uganda.^4^ Two example use cases highlight the robustness of DMsan for sanitation research, development, and deployment (RD&D): (i) use by decision makers interested in enhancing decision-making transparency and understanding the robustness of sanitation choices with uncertain and/or varying stakeholder input and technology ability and (ii) use by technology developers seeking to identify and expand the opportunity space for their technologies. These examples highlight novel capacity of DMsan to provide insight for sanitation decision making in individual contexts and to increase transparency in technology evaluations, RD&D, and deployment decision making.

## 2 Methods

### 2.1 Formulation of Decision-Making Framework

#### 2.1.1 Identification of Articles Focused on Decision Making in Resource-Limited Settings

To better understand the existing literature and techniques related to sanitation decision making, articles were gathered through Scopus using title, abstract, and keyword search terms that accounted for a variety of terms related to sanitation technologies (e.g., sanitation), resource-limited settings (e.g., low-income and marginalized), and decision making (e.g., decision making and multi-criteria; Table S2). The search was limited to papers published from 1960 through September 2020. The 1385 papers resulting from the initial search were screened for the inclusion criteria requiring that (i) they evaluated two or more sanitation technologies/systems, and (ii) the implementation context was in a resource-limited setting (Table S2). After screening, the remaining 35 papers were analyzed to summarize the decision-support techniques, criteria, and assumptions used in each paper, including the decision-support technique(s), stakeholder(s), criteria, sub-criteria, and indicators included in the analysis (Tables S3–S6, S19, S21, and S23). While we recognize that this focus on academic literature may not capture the full diversity of processes employed by decision makers overseeing the implementation of projects, it does serve as a starting point to ensure that the DMsan package has adequate flexibility to adapt to a wide range of end-user preferences.

#### 2.1.2 Justification of Decision-Making Methodologies, Criteria, and Indicators Included in the Decision-Making Framework

**Figure 1:**
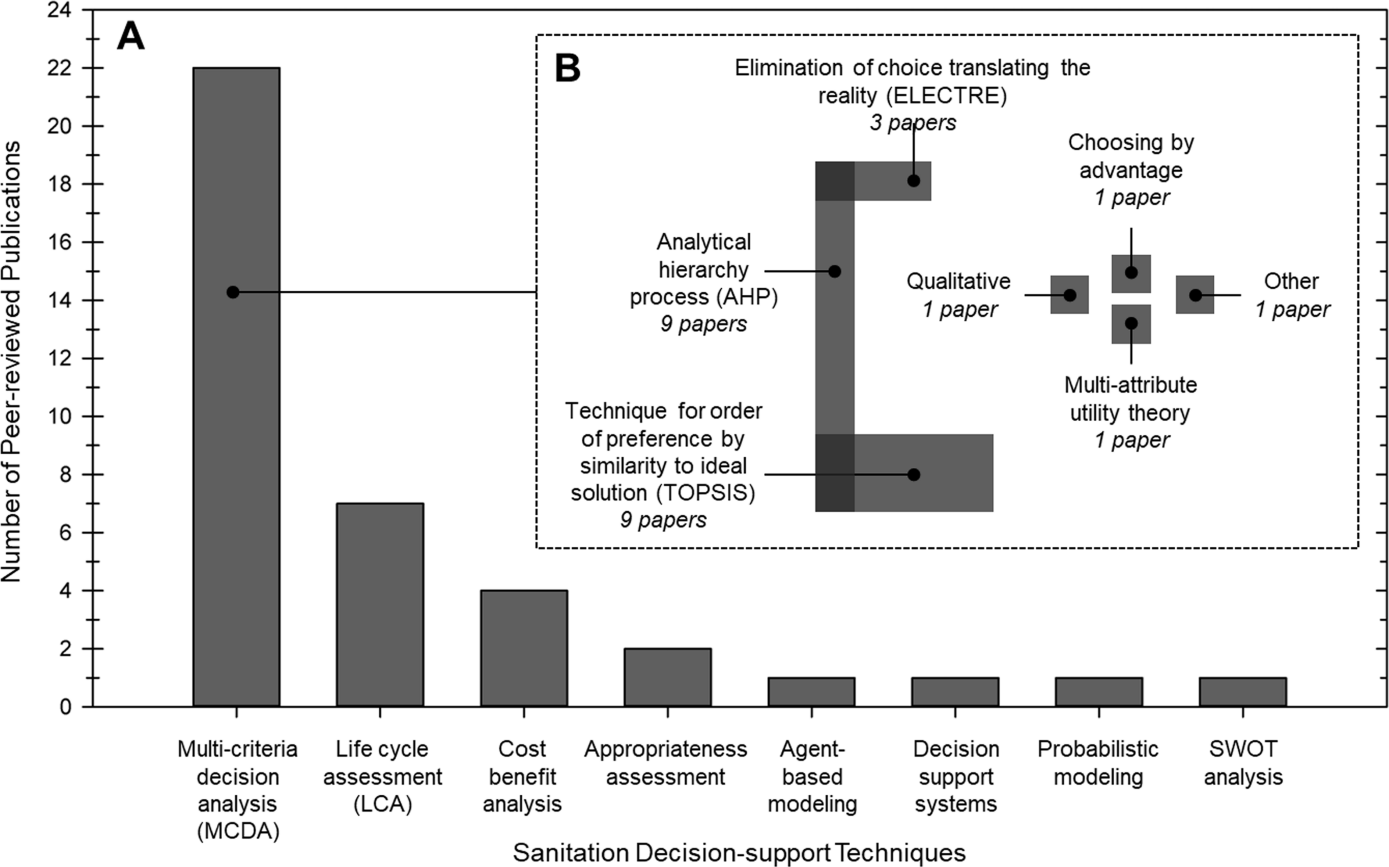
Literature review results used to develop the decision-making sanitation framework. (A) Summary of decision-support techniques in the peer-reviewed literature. The decision-support techniques most commonly used in sanitation decision making were multi-criteria decision analysis (MCDA) (22 of 35 total articles) and life cycle assessment (LCA) (7 of the 35 total articles). Other techniques included cost–benefit analysis (CBA); appropriateness assessment (AA); agent-based modeling; decision-support systems; probabilistic modeling; and strengths, weaknesses, opportunities, and threats (SWOT) analysis. (B) Breakdown of MCDA methods. The MCDA methods most used were the technique for order of preference by similarity to ideal solution (TOPSIS; 9 of 22 MCDA articles) and analytical hierarchy process (AHP; 9 of 22 MCDA articles).

The results of the literature review were used to inform the methodologies, criteria, and indicators used within the DMsan package. Decision-support techniques were identified for each paper, with some papers using multiple techniques.^13,27,28,31^ Out of the 35 papers, MCDA was the most common decision-support technique (22 papers)^3,10,13,25−28,31−45^ followed by LCA (seven papers).^12−14,27,28,31,46^ Other techniques included cost–benefit analysis (CBA; four papers);^47−50^ appropriateness assessment (AA; two papers);^51,52^ strengths, weaknesses, opportunities, and threats (SWOT) analysis (one paper);^53^ agent-based modeling (ABM; one paper);^54^ probabilistic model (one paper);^55^ and decision-support systems (one paper;^56^Figure 1A). Within MCDA, the most widely used ranking methodologies were the technique for order of preference by similarity (TOPSIS; nine papers)^13,25,27,28,31,32,38,40,41^ and analytical hierarchy process (AHP; nine papers;^10,26,28,33,34,37,38,43,44^Figure 1B). Other MCDA methods observed outside of TOPSIS and AHP included elimination of choice translating reality (ELECTRE; three papers),^26,35,36^ multi-attribute utility theory (one paper),^39^ qualitative MCDA (one paper),^42^ and choosing by advantage (one paper).^3^ MCDA methods were often combined to increase the robustness of the decision making framework by leveraging the advantages of multiple approaches, such as AHP paired with TOPSIS^28,38^ or AHP paired with ELECTRE.^26^ Some researchers argue that TOPSIS is a superior method because it yields multiple quantitative outputs,^9,11,12^ whereas AHP may be preferred because it generates criteria and indicator weights with consistent pair-wise comparisons (while TOPSIS requires subjective expert judgment for inputting weights).^10,33,34^

Through the literature review of commonly used criteria in sanitation and resource recovery decision making, four main criteria were identified: (i) technical or functional, relating to the system ability and function; (ii) environmental or ecological, representing the impacts a system inflicts on the environment; (iii) economic, associated with the economic costs of the system; and (iv) social or institutional, relating to technology adoption and social impacts (Table S3). Criteria and indicator definitions vary in literature, but for this work, criteria are defined as the main or principal decision-making categories and indicators are the specific aspects in which the technology alternative is assessed (Table S1). Environmental was the most widely used criterion, represented in 32 of the 35 papers. Within this criterion, common indicators used to assess the environmental impacts included energy consumption, air contamination, sludge production, water contamination, water consumption, odor, and eutrophication. The second most used criterion was economic (29 of 35 papers) that included operation and maintenance (O&M) costs, capital costs, and potential profit from resource recovery. The technical category was represented in 28 of 35 papers and included reliability, robustness, resiliency, complexity, resource recovery efficiency, land use, and flexibility of a system. Finally, the social and institutional criterion accounted for 26 of the 35 papers and included socio-cultural acceptability, job creation, and compatibility with local policy. Throughout the studies, resource recovery indicators were used to characterize technical, economic, environmental, and social criteria (e.g., quantity of resources, value of resources, and willingness to use resources). When developing the MCDA framework, resource recovery cost and environmental impact offsets (due to fertilizer and biogas production) were incorporated into the economic and environmental indicator calculations. However, the quantity of recoverable resources is important for contexts with limited access to resources or high resource users (e.g., significant agriculture producers); therefore, resource recovery was incorporated as a fifth criterion to capture quantity of resources recovered.

**Figure 2:**
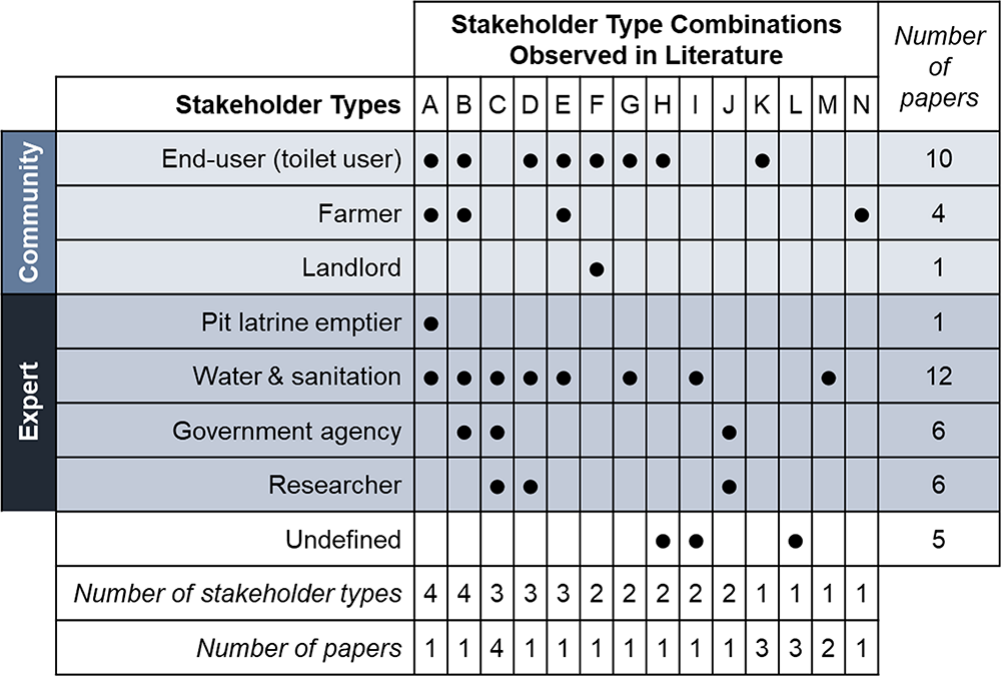
Summary of literature review of articles focused on decision making in resource-limited settings highlighting stakeholders involved in sanitation decision making. Each publication was characterized based on stakeholder engagement and the types of stakeholders engaged. Community stakeholder types include end-users (i.e., toilet users), farmers, and landlords. Expert stakeholder types include pit latrine emptiers, water and sanitation employees, government agencies, and researchers. The 14 unique stakeholder combinations were identified in literature (columns labeled A–N): A,^52^ B,^36^ C,^28,35,42,50^ D,^56^ E,^57^ F,^53^ G,^39^ H,^51^ I,^38^ J,^45^ K,^10,37,44^ L,^13,33,43^ M,^26,27^ and N.^46^ For example, one paper included stakeholders defined in combination A (end-users, farmers, pit latrine emptiers, and water and sanitation employees), and four papers included stakeholders defined in combination C (water and sanitation employees, government agencies, and researchers).

Although stakeholder engagement is important for successful deployment and sustainability of sanitation systems, stakeholder engagement with multiple stakeholder types was rare in the existing sanitation decision-making studies. Across the 35 publications examined in the literature review, only 22 publications mentioned stakeholder input. Stakeholder types included in the decision-making process were water and sanitation professionals (12 papers), end-users (10 papers), government agencies (six papers), researchers (six papers), farmers (four papers), pit latrine emptiers (one paper), and landlords (one paper), along with five papers that used non-specific “experts” to define a stakeholder (undefined; Figure 2). Papers varied from including one to four types of stakeholders. Incorporating feedback from multiple stakeholder types, depending on the decision being made, can greatly benefit decision making; however, time and resources often prevent significant stakeholder involvement. In particular, active stakeholder involvement in scenario modeling can support the setting of criterion and indicator weights based on community preferences. Decision makers and technology developers with access to stakeholder feedback can input stakeholder-informed criterion and indicator weights. Additionally, to enable decision makers and technology developers to generate insight despite limited access to stakeholder groups, DMsan was designed to run simulations across a complete spectrum of criteria and indicator weightings to identify contexts and stakeholder preferences that lead to the selection of a given technology to advance sustainable sanitation locally and globally.

### 2.2 DMsan MCDA Framework and Package Structure

#### 2.2.1 DMsan Overview

**Figure 3:**
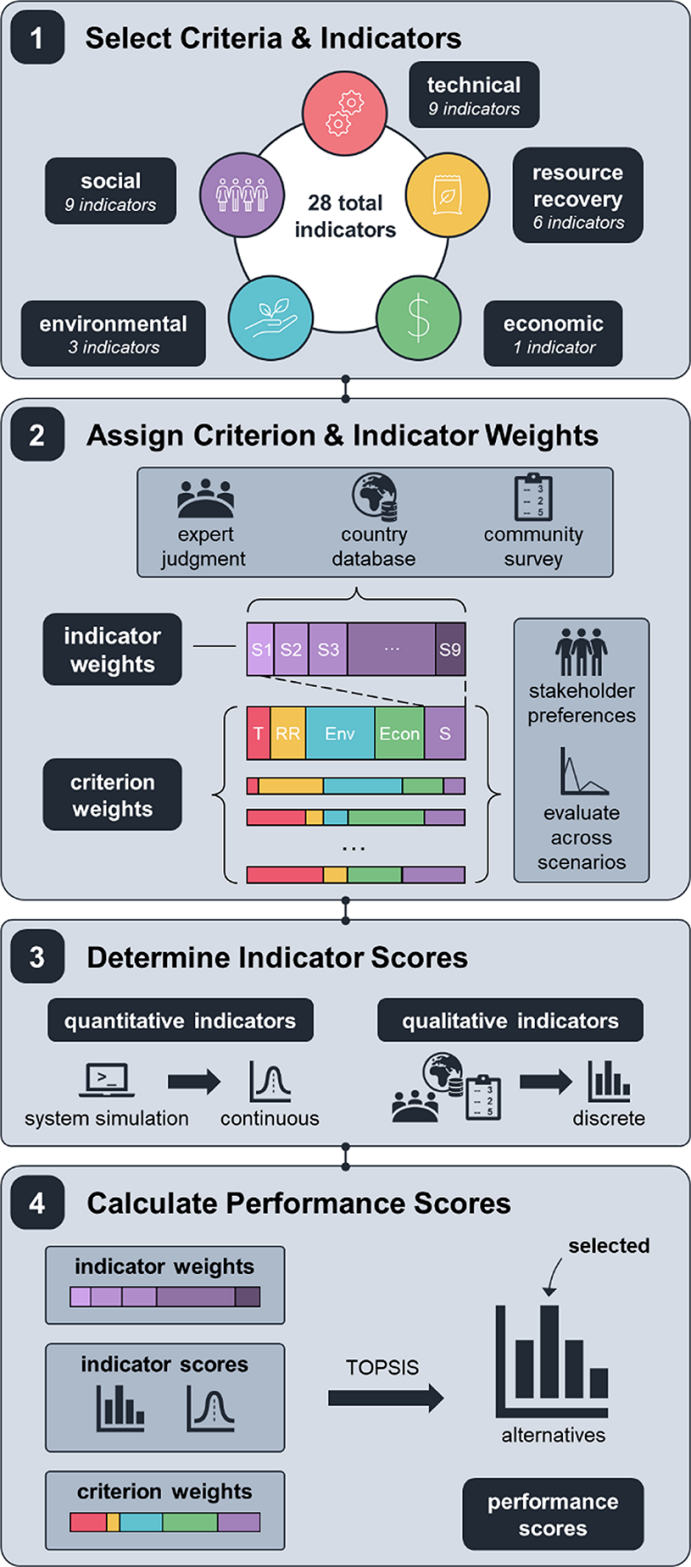
Overview of the MCDA methodology implemented in DMsan. The first step requires the user to select the relevant criteria and indicators to evaluate the sanitation alternatives for a specific context. Next, indicator weights are calculated using indicator contextual drivers for the country/territory through the AHP method, and criteria weights are either determined by community stakeholder preferences or generated by DMsan for the desired number of weight scenarios using Latin hypercube sampling. After the weights are established, indicator scores are assigned for each alternative: qualitative indicators are scored using predefined ranges (Section S2) and quantitative indicators are calculated through system simulation, TEA, and LCA in QSDsan. Finally, TOPSIS is used to calculate alternative rankings and performance scores to determine the best performing alternative for each criteria weight scenario.

Ultimately, the results of the literature review were used to inform the default MCDA methodologies and criteria included within the DMsan package: AHP paired with TOPSIS to evaluate the tradeoffs of five criteria (technical, environmental, resource recovery, economic, and social) characterized with 28 indicators (Table 1). DMsan was structured following the four key steps in MCDA: (i) selecting criteria and indicators, (ii) assigning weights to the criteria and indicators, (iii) determining indicator scores, and (iv) calculating performance scores of each alternative (Figure 3). To facilitate the execution of these steps, a Python (3.8+) package was developed, which contains generic algorithms and a built-in contextual driver database for rapid and flexible evaluation of the alternatives under uncertainty. Users can select criteria and indicators to include in an analysis from among the criteria and indicators included in the core structure or add new criteria and indicators as necessary. Criteria and indicators can be weighted using stakeholder informed weights, built-in criteria weight scenarios and a country/territory-specific contextual drivers database, or assumed weights. DMsan was constructed to enable integration with existing Python packages (e.g., QSDsan^29,30^) to simulate the quantitative indicator scores (for economics, environmental impacts, and resource recovery) of the system configurations with manual scoring for qualitative indicators (Section S5). QSDsan is an integrative platform for sanitation and resource recovery system design, simulation, techno-economic analysis, and life cycle assessment. QSDsan specifically can be used to calculate quantitative indicators, while DMsan is used to evaluate conflicting tradeoffs related to the alternatives’ indicator scores using MCDA. Sanitation decision makers and technology developers can access existing sanitation unit process models available on the public GitHub repository, and they can follow published tutorials to build new sanitation and resource recovery unit processes for use in DMsan. Alternatively, DMsan users can also manually input quantitative indicator score data without simulation in QSDsan if process modeling is unnecessary or data availability limits modeling capabilities. Overall performance of each alternative can be calculated via TOPSIS simulation default to DMsan. Source codes of the DMsan package are available as a public repository on GitHub,^58^ and the package has been released on the Python package index (PyPI) repository.^59^

**Table 1:** Summary of Criteria, Sub-Criteria, Indicators, and Indicator Contextual Drivers Included in the DMsan Package^b^

criteria	sub-criteria	indicator	indicator score type used in illustrative example^a^	contextual driver to determine indicator weight
technical	resiliency and robustness	user interface robustness	qualitative	extent of training^60^
		resiliency of treatment type	qualitative	population without at least basic sanitation^1^
	feasibility	accessibility to parts	qualitative	technology absorption^60^
		transportation feasibility	qualitative	quality of roads^60^
		construction skills required	qualitative	construction skills available^61^
		operation and maintenance skills required	qualitative	professional skills available^60^
	flexibility	population flexibility	qualitative	population growth rate^62^
		power outage flexibility	qualitative	electricity coverage^62^
		drought flexibility	qualitative	baseline water stress^63^
resource recovery	resource recovery feasibility	water recovery*	no indicator score	baseline water stress^63^
		nitrogen recovery	quantitative	nitrogen fertilizer fulfillment^64,65^
		phosphorous recovery	quantitative	phosphorous fertilizer fulfillment^64,65^
		potassium recovery	quantitative	potassium fertilizer fulfillment^64,65^
		energy recovery	quantitative	renewable energy consumption^62^
		supply chain feasibility	qualitative	infrastructure quality^60^
environmental	life cycle environmental impacts	damage to ecosystems	quantitative	indicator weights are distributed equally across LCA categories^66^
		damage to human health	quantitative	
		damage to resources	quantitative	
economic	net life cycle costs	annual cost per capita	quantitative	no indicator weight
social	job creation	total jobs created	quantitative	unemployment rate^62^
		high-paying jobs created	quantitative	international poverty line^61^
	end-user acceptability	disposal frequency	quantitative	determined by end-user community survey^4^
		cleaning requirement	qualitative	
		privacy	quantitative	
		odor and flies	qualitative	
		security	quantitative	
	property manager acceptability	disposal frequency*	no indicator score	determined by property manager community survey^4^
		cleaning requirement*	no indicator score	

a The indicator types shown in the table describe the designations used in the illustrative example of this manuscript. It should be noted that decision makers and technology developers using DMsan have complete flexibility to choose how to quantify indicators (quantitative vs qualitative) to best suit the goals and data availability for their analysis.

b Sub-criteria were developed for the conceptual organization of criteria into indicators and are not assigned scores or weights. Qualitative and quantitative indicator scores (highlighted in the column “indicator”) are assigned and calculated for each sanitation and resource recovery system alternative (Section S2). Indicator contextual drivers are used to calculate indicator weights within a criterion (highlighted in the column “contextual driver to determine indicator weight”; Section S3). Indicators with an asterisk (*) were excluded in the illustrative example because property managers were not responsible for system disposal and cleaning efforts, and none of the systems incorporated water recovery.

#### 2.2.2 Criteria and Indicator Selection

Informed by the literature review, DMsan includes five criteria commonly used in sanitation decision making to evaluate the capability and sustainability of sanitation and resource recovery systems. The criteria include technical (i.e., the engineering design requirements), resource recovery (i.e., the ability of the system to recover nutrient, energy, and water resources for reuse), environmental (i.e., the life cycle environmental impacts), economic (i.e., the life cycle costs per capita), and social (i.e., the acceptability for the system users and operators; Table 1). Each criterion is divided into one or more sub-criteria to further describe its contribution to decision making: resiliency and robustness, feasibility, and flexibility (technical); resource recovery feasibility (resource recovery); life cycle environmental impacts (environmental); net life cycle costs (economic); and job creation, end-user acceptability, and property manager acceptability (social). It should be noted that sub-criteria were developed for conceptual organization of the criteria and indicators and do not have sub-criteria scores or weights assigned. The sub-criteria are matched with 28 qualitative and quantitative indicators to represent the capability of each system by rating on a predefined scale or quantitative calculations (Section S2). In evaluation, DMsan users can choose to include all or any combination of the five criteria and 28 indicators available in the package or to add and/or modify criteria or indicators as desired. This flexibility is important because it can accommodate decision-making methods and indicators that were not presented in peer-reviewed papers (e.g., institutional reports) or that are specific to a certain community.

#### 2.2.3 Criteria and Indicator Weight Assignment

The second step is to assign criteria and indicator weights (Figure 3). Weighting within DMsan is conducted in two phases. Criterion weight^26^ is assigned to each criterion and represents the relative importance of that criterion in the decision-making process, and indicator weight^26^ is assigned to each indicator and represents the importance of that indicator, within its criterion, in a given context. Criterion weights are used to reflect stakeholder preference, and users of DMsan can manually input a weight for each criterion (technical, resource recovery, environmental, economic, and social) when community preference data is available or if they wish to evaluate a specific combination of criterion weights. In lieu of such data, users can specify the desired number of criteria weight scenarios (e.g., 1000) and DMsan will generate a set of criterion weights using Latin hypercube sampling (Figure S1) to evaluate the entire spectrum of weight options for each criterion (criterion weight ranges from 0 to 1).

Indicator weights are used to rate the relative importance of each indicator’s contribution to a system’s overall criterion score for each of the five criteria (Section S3). Weights can be determined by using (i) relevant stakeholder surveys employing AHP, (ii) an embedded database that estimates the relative importance of each indicator in a given country/territory, or (iii) assumptions when data is unavailable for the basis of indicator weights (e.g., assumed equal distribution of weights for environmental indicators).

AHP was selected to calculate indicator weights, informed by community and stakeholder surveys, due to its use in multiple fields of environmental science^18^ and its ability to calculate weights based on simple pair-wise comparisons. Community and stakeholder surveys can be used to collect data on pair-wise preference between indicators (e.g., indicator A is moderately more important than indicator B). In the absence of data on stakeholder’s pair-wise comparisons between indicators, DMsan users can utilize a database of country/territory-specific preferences (indicator contextual drivers) to inform pair-wise preferences. It is assumed that the relative importance of a contextual driver (on a scale of 0–100) for a country/territory is equivalent to the relative importance of its indicator. A technical indicator example was technology absorption (i.e., a country’s absorption of the latest, most novel technology), which was selected as the basis for the indicator weight for accessibility to parts. Countries with higher levels of advanced technology could have better access to custom parts required for novel and advanced systems—resulting in a low importance of accessibility to parts because all parts are viewed as accessible. As a resource recovery indicator example, the indicator weight for nitrogen recovery is calculated using the need for additional nitrogen fertilizer in the country calculated as the ratio between the mass of nitrogen fertilizer used by the country and the recommended mass of nitrogen fertilizers for a country’s crop production. Countries with ratios closer to 0 indicate a clear need for nitrogen-based fertilizers (resulting in a higher indicator weight for nitrogen recovery), which could be met through production of human excreta-derived fertilizers. The database consists of compiled data produced by the World Economic Forum,^60^ the World Health Organization and United Nations Children’s Fund,^1^ the International Labour Organization,^61^ the World Bank,^62^ the World Resources Institute,^63^ and the Food and Agriculture Organization of the United Nations.^65^ The database does not contain contextual driver data for environmental, economic, or end-user/property manager acceptability indicators. Users can conduct community surveys or make assumptions when data is unavailable.

#### 2.2.4 Indicator Score Determination

Quantitative resource recovery, environmental, and economic indicator scores can be determined by conducting system simulations, TEA, and LCA in QSDsan^29,30^ or by manually entering scores calculated outside of QSDsan. When using QSDsan, the quantities of recoverable nitrogen, phosphorus, potassium, and energy are calculated based on country-specific dietary intake parameters, expected nutrient and carbon excreted,^64,67,68^ and technology-specific nutrient and energy recovery efficiencies.^64,67,68^ The quantity of recoverable water is calculated as the volume of treated water that can be used for potable or non-potable uses. TEA is used to calculate the annual cost per capita for each sanitation system alternative. LCA calculations use the life cycle impact assessment method ReCiPe to calculate three endpoint environmental indicators (damage to human health, damage to ecosystems, and damage to resource availability) with options to select individualist, hierarchist (default method), and egalitarian cultural perspectives for the calculation.^66^ Both TEA and LCA calculations incorporate context-specific input parameters (e.g., material costs, electricity prices, and electricity source) to quantify location-specific costs and environmental impacts. Monte Carlo analysis with Latin hypercube sampling^69^ is used to evaluate the impact of uncertainty in modeling inputs on quantitative indicator scores (i.e., annual cost per capita, quantity of recovered resources, environmental impact indicators, and job creation; Section S5). Qualitative indicator scores related to the technical, resource recovery (i.e., supply chain feasibility), and social criteria (i.e., end-user and property manager acceptability indicators) are assigned using predefined score ranges (e.g., user interface robustness score ranges from 1 to 5 depending on the complexity of the toilet; Table S7). Because systems are a combination of several sanitation unit processes, the system scores—using predefined score ranges—are assumed to be the score of the worst performing unit process within the system (e.g., a system composed of both anaerobic and chemical/thermal treatment would receive a resiliency of treatment type score that of anaerobic treatment, which had higher maintenance needs; Table S8).

#### 2.2.5 Performance Score Calculation

Sanitation system performance scores indicate the ability of a specific sanitation system to outrank the other alternatives (i.e., a higher score indicates better system performance). Because TOPSIS has a stronger mathematical foundation for quantitatively viewing tradeoffs among criteria compared to other ranking methodologies,^27,31,70^ it was selected to calculate performance scores and ranks in DMsan using the criteria weights, indicator weights, and indicator scores (Section S4).

### 2.3 Illustrative Applications of DMsan for Decision Makers and Technology Developers

#### 2.3.1 Bwaise, Uganda Context, and Sanitation System Alternatives

To illustrate the utility of DMsan, we evaluated sanitation and resource recovery systems for Bwaise, an informal settlement in Kampala, Uganda, described by Trimmer et al.^4^ Bwaise is located in northern Kampala and is rapidly growing with over 100,000 people. Although sanitation is reported as a high development priority among residents, systems typically fail due to limited stakeholder participation.^9^

**Figure 4:**
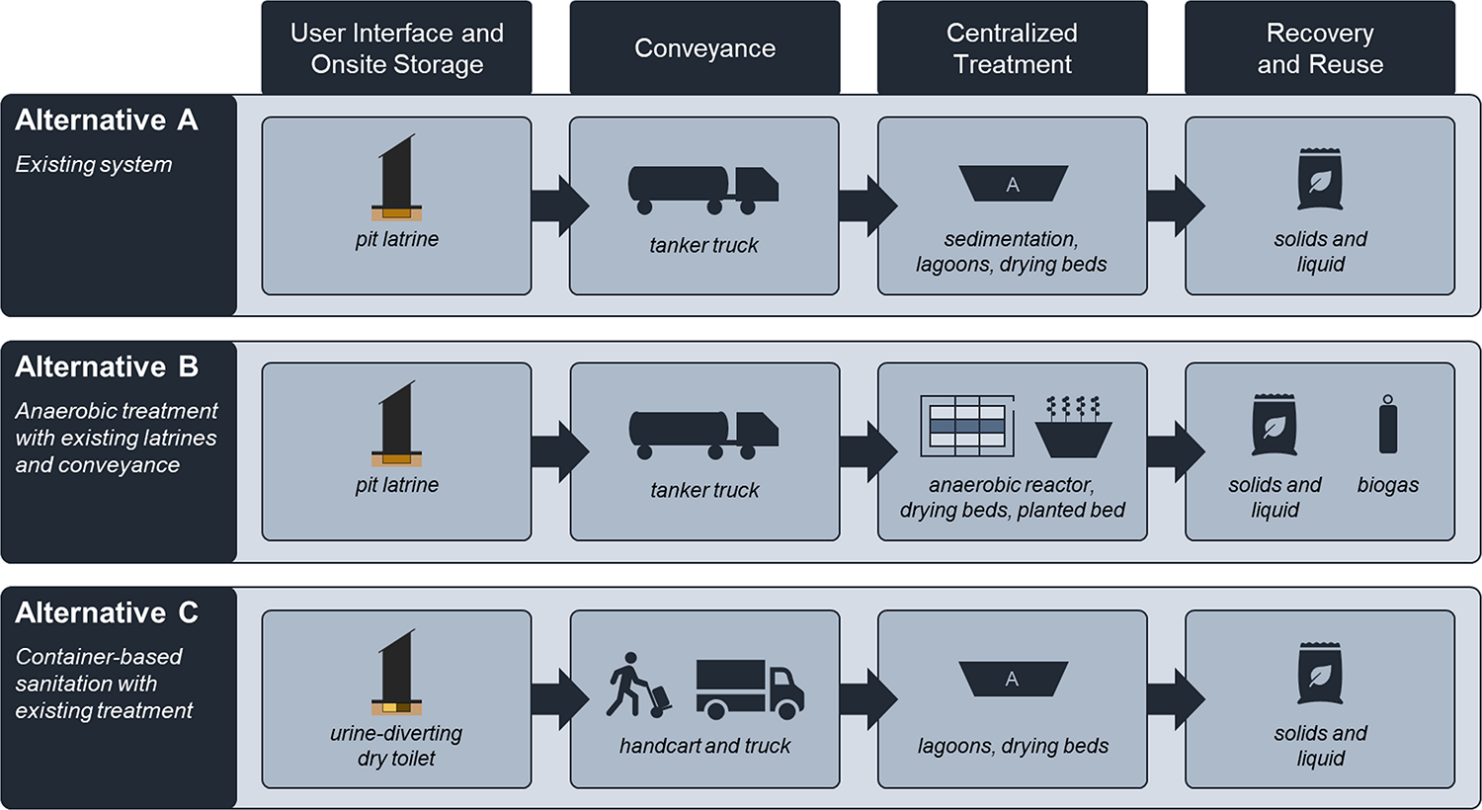
Overview of the Bwaise, Uganda sanitation system alternatives as described by Trimmer et al.^4^ Each alternative incorporates a user interface with onsite storage, conveyance, centralized treatment, and recovery and reuse. Alternative A is the existing system, whereas Alternative B replaces the centralized treatment system, and Alternative C leverages source separation and alternative conveyance. All three alternatives are described in more detail in the text.

Three sanitation system alternatives were evaluated based on the systems described by Trimmer et al.^4^ All alternatives incorporated a user interface, storage, conveyance, centralized treatment, and recovery of nutrients and/or biogas (Figure 4). The first alternative (Alternative A) is the existing sanitation system incorporating pit latrines, vacuum collection trucks, centralized treatment (sedimentation, solids drying beds, and lagoons), and recovery of nutrients for fertilizer (dried solids and nutrient-rich liquid effluent). Alternative B replaces the existing centralized treatment with an anaerobic baffled reactor, solids drying beds, and an additional planted bed for liquid treatment with solid and liquid nutrients recovered for land application and biogas for cooking fuel. Alternative C replaces the existing pit latrines with container-based urine-diverting dry toilets that use urine handcart and solids truck transportation to bring resources to the centralized treatment facility described in Alternative A, excluding sedimentation, as liquids and solids are already separated. Alternative C increases the nutrient recovery potential (relative to Alternatives A and B) through source separation.

All five criteria and 26 of the 28 indicators were used in this illustrative analysis. The two excluded indicators were related to property manager acceptability (disposal frequency and cleaning requirement); they were excluded because they were not applicable since property managers were not responsible for system disposal and cleaning efforts. The two indicators related to disposal frequency and cleaning requirement by end-users were maintained to account for end-user responsibilities. Each alternative was simulated using the module developed in QSDsan^29,30^ to calculate quantitative scores for resource recovery (quantity of recovered nitrogen, phosphorus, potassium, energy, and water), environmental (damage to human health, damage to ecosystems, and damage to resource availability), and economic (annual cost per capita) indicators (Section S5). Annual user cost and life cycle environmental impact estimates incorporated construction and operation of onsite and centralized facilities, conveyance, direct emissions from excreta degradation, and income and environmental impact offsets from recovered products.^4^ Monte Carlo analysis with Latin hypercube sampling (1000 simulations) was used to account for possible variations in the QSDsan input parameters. Scores for qualitative technical, resource recovery, and social indicators were assigned based on each alternative’s capability on a predefined scale (Section S2).

To avoid constraining insight to a limited set of stakeholder-informed criteria weights, 1000 criteria weight scenarios were generated using DMsan’s embedded algorithms to represent the entire landscape of stakeholder preferences (Figure S1). Indicator weighting incorporated end-user community surveys, Uganda-specific contextual drivers found in the country/territory-specific database, and assumptions. Technical, resource recovery, and job creation social indicators were weighted using the values for Uganda in the country/territory-specific contextual driver database. The end-user community survey results presented by Trimmer et al.^4^ were used to calculate the indicator weights related to social end-user acceptability (Section S2.5.2). Uniform weights were used for environmental indicators (i.e., 1/3 weight for each of the three indicators), and economic does not need an indicator weight because it only has one indicator (annual cost per capita). All codes used in the illustration are publicly available on GitHub.^59^

#### 2.3.2 Use of DMsan by Decision Makers: Navigating the Choice among Alternatives

DMsan can be used by a wide range of stakeholders interested in assessing the sustainability and performance of sanitation and resource recovery system alternatives. The first illustration of the use of DMsan focuses on the implementation decision makers (e.g., sanitation engineers, urban planners, and utility staff) selecting a sanitation system among alternatives. The illustration highlights the utility of DMsan using a built-in indicator and criteria weight scenarios in the absence of community-informed preferences. MCDA results are presented for each alternative as the “probability of having the highest performance score (i.e., winning)” and the “scenarios in which the alternative has the highest winning probability”. For each criteria weight scenario, the probability of an alternative winning was calculated (the probability was based on the uncertainty in QSDsan inputs) as the ratio between the total number of times it has the highest performance score among all alternatives and the total number of indicator uncertainty simulations (1000). If an alternative has a higher winning probability than other alternatives (even if it is only by 1%) in a specific criteria weight scenario, then the criteria weight scenario will be included in an alternative’s opportunity space. Decision makers can use these results to navigate the choice among alternatives under varied stakeholder priorities and understand how criteria importance influences the selection.

#### 2.3.3 Use of DMsan by Technology Developers: Expanding the Opportunity Space of Select Alternatives

The second illustration of the use of DMsan focuses on the technology developers interested in expanding the opportunity space of their technology. Technology developers can use DMsan to explore how improvements to specific indicators (e.g., decrease annual cost per capita and increase energy recovery) can impact a system’s sustainability and opportunity space. Technology developers can identify which indicators can be reasonably improved. In this illustration, indicator scores were modified from the baseline value to the theoretical best score, and technology developers can observe how specific indicator improvements impact the percent of criteria weight scenarios that their technology has the highest performance score compared to the baseline opportunity space. This illustration also provides a means for the technology developers to identify a path forward for technology development by evaluating the effect of simultaneous indicator improvements.

## 3 Results of the Illustrative Examples and Discussion

### 3.1 Insight for Decision Makers: Understanding Stakeholder Influence on MCDA Outcomes

**Figure 5:**
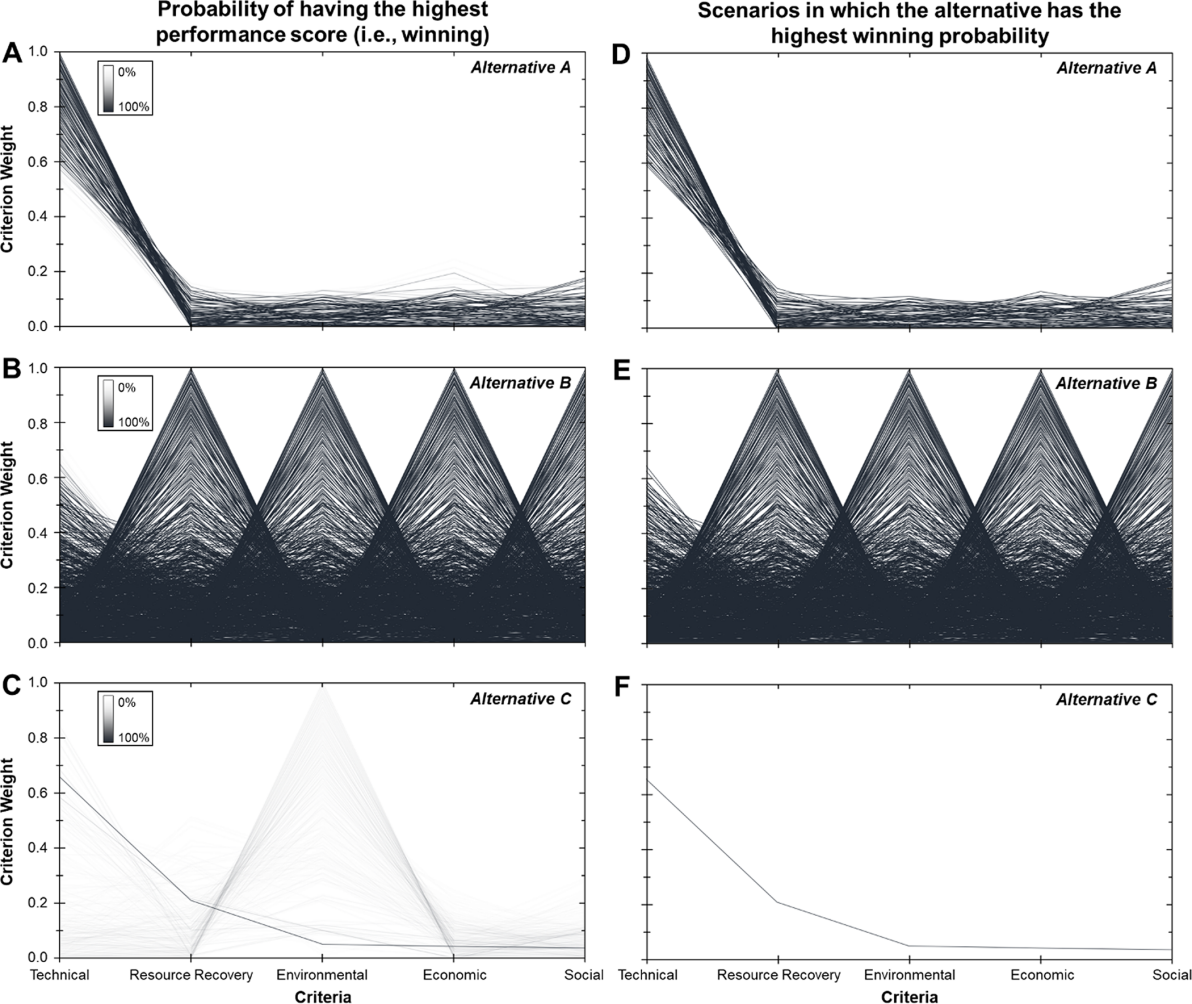
Evaluation of alternatives under varied criteria weight scenarios for decision making. (A–C) Probability of having the highest performance score among alternatives (0–100%). Each line represents a single criteria weight scenario, with darker lines indicating higher probabilities. (D–F) Criteria weight scenarios in which an alternative has the highest winning probability across the three alternatives. A line for a particular criteria weight scenario is present if the alternative is the best performing alternative. Among 1000 criteria weight scenarios, Alternative A outperforms B and C in 77 scenarios (shown in panel D), Alternative B outperforms A and C in 922 scenarios (shown in panel E), and Alternative C outperforms A and B in one scenario (shown in panel F). Alternative A is the best performing system when stakeholders place high importance on technical ability (criterion weight ∼0.6 to 1.0). The probability of the highest performance score increases for Alternative A as the technical criterion weight approaches 1. Alternative B has the highest performance score in criteria weight scenarios with high criterion weights for resource recovery, environmental, economic, or social or when criteria weights are evenly distributed. Alternative C is unlikely to be selected as the best performing alternative without improvements across indicators.

From the sanitation decision maker perspective, the results of DMsan can be used to identify the best alternative for implementation contexts with specific community preferences (i.e., criteria weight scenarios). For example, Alternative A (existing system) would be selected in 77 of the 1000 criteria weight scenarios, Alternative B (biogas recovery system) would be selected in 922 out of 1000 scenarios, and Alternative C (source separation system) would be selected in 1 of the 1000 scenarios (Figure 5). Although Alternative B outperforms Alternatives A and C in 92.2% of the criteria weight scenarios, each alternative still has an opportunity to be selected for implementation in the community, depending on the relative importance of the five criteria as perceived by community members and stakeholders during decision making. Decision makers can use these results to understand what community preferences lead to a particular technology’s selection and identify communities best suited for implementation based on the importance placed on decision-making criteria. For example, Alternative A is likely to be selected in communities that place the highest importance on the technical criterion (with technical criterion weights between 0.6 and 1.0; Figure 5A,D), while Alternative B is likely to be selected almost all criteria weight scenarios when the technical criterion is weighted less than 0.6. This finding stems from the fact that Alternative A outperforms or matches the capability of Alternative B in all technical indicators. It should be noted that Alternative C outperforms or meets the performance of Alternative A in nine of the nine technical indicators, but the relative importance of each indicator weight led to Alternative A being the best within the technical criterion. For example, Alternative C outperforms Alternative A in flexibility to power outages, but the indicator only contributes 2% to the overall technical criterion for decision making due to the infrequent nature of power outages in Uganda compared to the global maximum. Sanitation decision makers may find Alternative A sustainable when technical factors (i.e., simple user interface, minimal construction, and maintenance skills required) are highly favored within the community.

The opportunity space for Alternative B is expansive as it scores well in recovering energy and nutrients, produces lower environmental impacts, and generates high economic returns from biogas recovery (Figure 5E). It also fosters end-user acceptability with the simple pour-flush toilet design to minimize odors, cleaning, and maintenance while also creating jobs for the community. However, Alternative B does not outrank other alternatives when technical criteria are favored with weights higher than 0.6. As the technology criterion weight approaches 0.6, the probability of Alternative B having the highest performance score decreases and eventually approaches zero because it is outperformed across technical indicators (Figure 5B). This system may not be an appropriate alternative for communities that value accessible parts, simple construction skills, and minimum operation and maintenance skills. Sanitation decision makers should target implementation of Alternative B in communities that value resource recovery (i.e., phosphorus, potassium, and energy), environmental, economic, and/or social factors.

Alternative C has the highest likelihood of outperforming Alternatives A and B in one criteria weight scenario where community members have some preference toward technical and resource recovery capabilities (Figure 5F). When considering the uncertainty in indicator scores, Alternative C can outperform Alternative B when environmental indicators are valued as Alternative C has lower damage to ecosystems and human health than Alternative B (Figure 5C). In the case of selecting one technology over another, sanitation decision makers should also consider the magnitude of differences in the simulated performance scores among the alternatives to determine if the differences are enough to choose one alternative over the others. Overall, these results can guide decision makers in selecting the most appropriate alternative for communities with varied decision-making preferences.

### 3.2 Insight for Technology Developers: Expanding the Opportunity Space through Indicator Improvements

**Figure 6:**
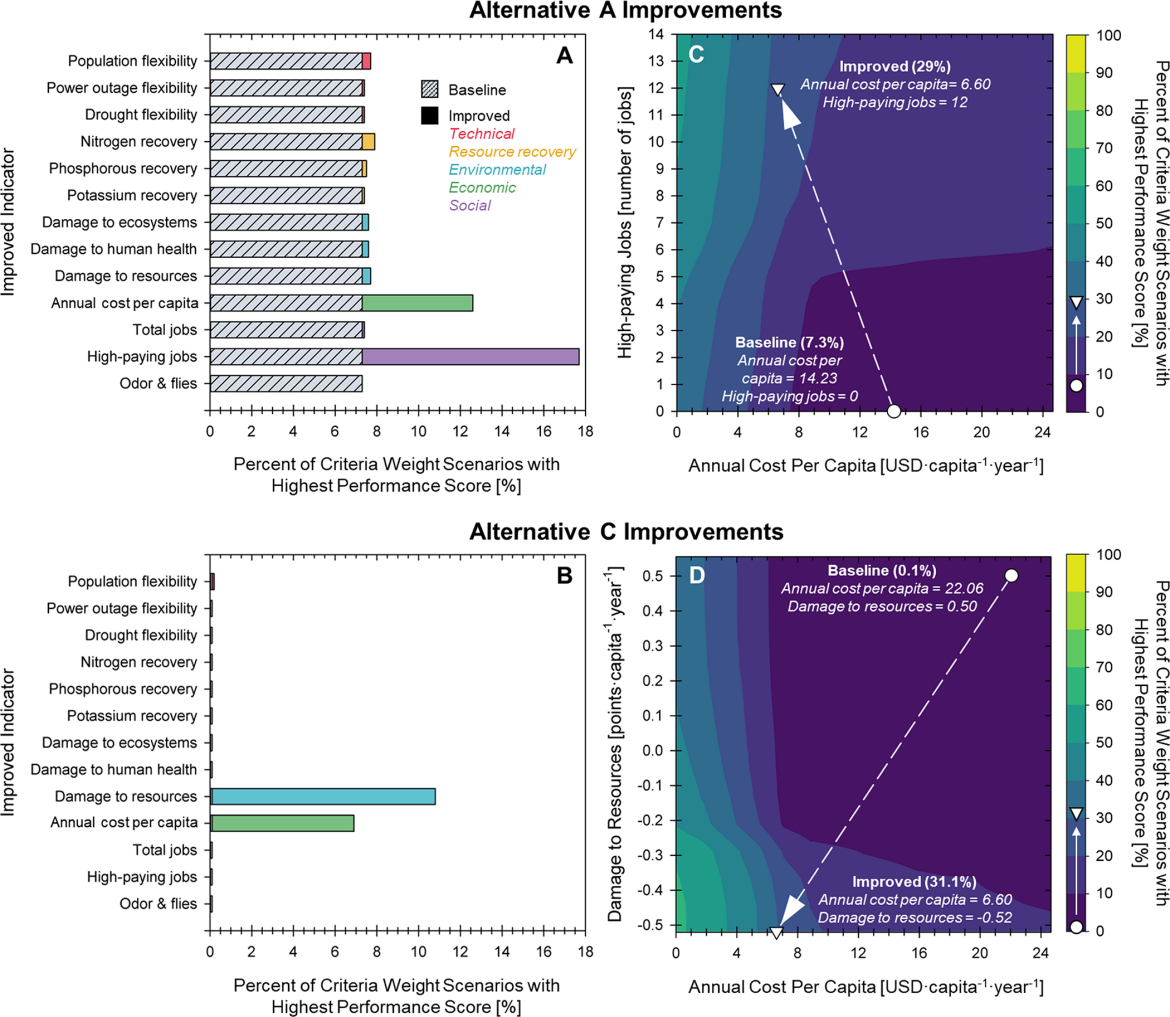
Indicator improvements to increase opportunities for selection of (A, C) Alternative A and (B, D) Alternative C. (A, B) Individual impact of indicator improvements on the percent of criteria weight scenarios for which Alternatives A and C have the highest performance score. Overall, 13 indicators were individually improved to the best indicator score to evaluate its individual impact on the alternative’s performance. Economic and environmental indicators were set to 10%, better than the best indicator score among the three alternatives (e.g., the improved economic score was 6.60, which is 10% lower than the least expensive alternative, Alternative B). Indicators were included in the analysis if they could reasonably be improved without requiring a complete design change (e.g., adding jobs does not require inherent changes to the technology, while energy recovery requires significant design changes to Alternatives A and C). (C, D) Combined impact of the top two indicator improvements on the percent of criteria weight scenarios for which Alternatives A and C have the highest performance score. Altogether, technology developers can use these results to prioritize research and development to achieve indicator improvements that would expand their technology’s opportunity space.

DMsan can also be used by technology developers to identify indicator improvement opportunities that lead to the technology’s selection in the criteria weight scenarios in which it was initially outperformed by other alternatives. Since Alternative B outperforms the others in 92.2% of the 1000 criteria weight scenarios, Alternatives A and C were considered in the indicator improvement analysis. Indicators were included in the analysis if they reasonably could be improved without requiring a significant technology redesign. For example, the indicator resiliency of treatment type was excluded from the analysis because improvements to that indicator require a completely different treatment type category for the alternative (e.g., moving from an anaerobic to a chemical treatment system); however, the indicator annual cost per capita was included because technology developers could investigate ways to reduce costs without significantly changing the design of the system. As a result, of the 26 indicators analyzed, 13 indicators were selected for the indicator improvement analysis (i.e., three technical, three resource recovery, three environmental, one economic, and three social; Figure 6). Each indicator score was modified from the baseline value (i.e., median value in the indicator score uncertainty analysis) to the theoretical best score. For quantitative indicators, the theoretical best score was set to 10% better than the median indicator score of the best scoring alternative for the indicator (e.g., the theoretical best score for annual cost per capita was set to 6.60 USD·capita^–1^·year^–1^, which was 10% lower than Alternative B’s median of 7.34 USD·capita^–1^·year^–1^ annual cost per capita). This ensured that the improved alternative could outperform the other alternatives for each quantitative indicator. For qualitative indicators, the theoretical best score was set to be the top score identified in the predefined score ranges (e.g., if scored on a 1–5 scale, then the top score is 5; Section S2). Although some indicators may be more difficult to improve than others, understanding which indicators drive increased selection of an alternative and how far an indicator must be improved to reach a desired percent of criteria weight scenarios can aid technology development.

At its baseline, Alternative A outperformed Alternatives B and C in 7.3% of the criteria weight scenarios. Increasing the quantity of high-paying jobs from 0 to 12 and decreasing the annual cost per capita from 14.23 to 6.60 USD·capita^–1^·year^–1^ resulted in the greatest impact on performance across criteria weight scenarios, which increased Alternative A’s opportunity space from 7.3% of criteria weight scenarios to 17.7 and 12.6%, respectively (Figure 6A). The remaining 11 indicator improvements lead to a marginal impact on the percent of criteria weight scenarios where Alternative A has the highest performance score (7.3–7.9% criteria weight scenarios). For Alternative C, indicator improvements with the most impact on its baseline performance were decreasing the damage to resources from 0.50 to −0.52 points capita^–1^·year^–1^ and decreasing the annual cost per capita from 14.23 to 6.60 USD·capita^–1^·year^–1^ (Figure 6B). These two improvements resulted in a notable increase in performance of Alternative C across the criteria weight scenarios (from 0.1 to 10.8 and 6.9%, respectively). Of the remaining 11 indicators, improving population flexibility resulted in increased performance for Alternative C (0.2%), while changes to the other 10 did not affect performance. Technology developers can use these results to develop a path forward for improving indicators that limit the implementation of their system. It is important to note that technology developers will need to know which changes are realistic for the alternative to maximize the value of the test to inform research and development.

While analyzing individual impacts can help to understand how indicators can improve an alternative’s performance, the effect could be more significant if multiple indicators are improved at the same time. The effect of changing a given alternative’s top two indicators simultaneously was evaluated for both Alternative A (high-paying jobs and annual cost per capita) and Alternative C (damage to resources and annual cost per capita). Annual cost per capita was varied from 0 to 24.65 USD·capita^–1^·year^–1^ (10% higher than the highest annual cost per capita among the three alternatives), high-paying jobs was varied from 0 to 14, and damage to resources was varied from −0.52 to 0.56 points·capita^–1^·year^–1^ (+/–10% of the best and worst scores among the three alternatives). When evaluating the spectrum of indicator improvements, technology developers can see how far an indicator must be improved to reach a desired percent of criteria weight scenarios that the alternative has the highest performance score. For example, in the case of Alternative A, decreasing the annual cost per capita to 14.23 USD·capita^–1^·year^–1^ and increasing the number of high-paying jobs to 12 can increase the opportunity space of Alternative A from 7.3 to 29% of criteria weight scenarios (Figure 6C). It should be noted that increasing the number of high-paying jobs will increase the annual cost per capita, but these indicators were varied independently for this illustrative analysis in Figure 6C. In reality, however, under the study’s set of assumptions, each additional high-paying job increases the annual cost per capita by 0.03 USD·capita^–1^·year^–1^ and by 0.41 USD·capita^–1^·year^–1^ for 12 additional jobs. Likewise, the impact of combining reductions in annual cost per capita with reductions in damage to resources was evaluated for Alternative C (Figure 6D). At its baseline, Alternative C outperforms Alternatives A and B in one criteria weight scenario (0.1% of scenarios), but with combined indicator scores identified in the indicator improvement analysis, it can increase performance to be selected in 31.1% of the criteria weight scenarios. To reduce the damage to resources (calculated via LCA), technology developers can identify the features of the technology driving environmental damage (e.g., electricity requirements and materials) and evaluate strategies to mitigate impacts such as transitioning to renewable energy sources, using more sustainable materials, or by increasing quantity of resources recovered (thereby increasing recovered resource offsets). Although not included in DMsan outputs, individual impact indicator values for a given LCIA method as well as the complete breakdown of LCA impacts (and costs and treatment performance) by the sanitation unit process or life cycle stage can be observed via QSDsan simulations of the alternatives. If technology developers are unable to reduce damage to resources, then cost reductions could still be a pathway to increase the selection of Alternative C. Technology developers could seek out grants and subsidies to offset the annual cost per capita further. Identification of indicator improvements to increase an alternative’s selection is only the first step toward implementing improvements. Although outside of the scope of the results presented in this study, technology developers should conduct further analyses to identify the pathway to implement the desired improvement (e.g., from minor technology design modifications up to significant policy changes). Overall, technology developers can use DMsan to identify the indicators driving their system’s performance and investigate how any changes in indicator scores may affect their technology’s opportunity space for implementation.

### 3.3 Implications of MCDA Framework and DMsan for RD&D of Sanitation and Resource Recovery Technologies

In this work, an MCDA framework was synthesized with well-defined indicators and criteria commonly used by sanitation and resource recovery decision makers. The framework incorporates robust decision-support techniques (e.g., LCA and TEA) to evaluate the performance of a portfolio of technologies and defines contextual drivers that can be used to calculate indicator weights (as a starting point) in the absence of stakeholder input. Incorporating the MCDA framework, DMsan was developed as an open-source package that enables users to transparently compare sanitation and resource recovery alternatives and evaluate the opportunity space for technologies. This package can help expedite the research and development of technologies by incorporating a comprehensive assessment of their performance related to technical, resource recovery, environmental, economic, and social drivers of decision making. DMsan is the first step in assisting technology developers that lack the resources (e.g., time, access to diverse groups of stakeholders, availability of high-resolution temporal and spatial data, and travel to deployment sites) during the technology’s research and development stage. DMsan is designed to easily integrate with other open-source packages (e.g., QSDsan^30^) and allows developers to mix and match technologies or unit operations in the sanitation resource chain (i.e., user interface, storage, conveyance, treatment, and distribution of resources) and seamlessly add or eliminate decision-making criteria and indicators as needed. Ultimately, technology developers can use the package to model stakeholder preferences and target deployment in contexts with similar stakeholder preferences and contextual drivers that lead to their technology outperforming other alternatives. DMsan can be used to assess preliminary sustainability for alternatives with built-in criteria weight scenarios.

For sanitation decision making focused on deployment, localized stakeholder preferences should be included in the package from multiple community and expert groups relevant to the decision (water and sanitation professionals, end-users, government agencies, farmers, etc.). Because the indicators included in the package were selected based on published peer-reviewed literature, DMsan users should consider surveying sanitation and resource recovery decision makers and practitioners in settings relevant to their work, which could help identify and incorporate useful indicators that were not included in traditional academic distribution channels (e.g., community-specific indicators related to governance and institutions).

In its current state, DMsan is ready to be used by decision makers and technology developers familiar with quantitative sustainable design^24^ and the programming language Python. However, future work is needed to develop a graphical user interface that eliminates the need for end-users to develop codes; this additional feature could expand the user base by making it more accessible to non-technical community decision makers. Future DMsan users should be engaged during the user interface development process to ensure successful, sustained use. Overall, DMsan overcomes the challenges presented in existing MCDA tools that prevent the evaluation of the opportunity space of new technologies. DMsan accommodates multiple criterion and indicator weighting methods (e.g., leveraging a database for contextual drivers) compared to existing tools that include limited weighting scenarios that constrain insight (e.g., customary or literature-informed weights vs context-specific stakeholder-informed weights). Additionally, instead of using fixed inputs for quantitative indicators, DMsan incorporates a robust uncertainty analysis workflow that allows all feasible indicator values to be used in decision making to develop an opportunity space of potential decisions instead of a single result. Ultimately, DMsan provides the field of sanitation and resource recovery a valuable decision-making tool to evaluate a technology’s opportunity space with variable stakeholder input to guide deployment and increase access to and the sustainability of sanitation.

## Supporting Information Available

Terminology definitions; literature review of decision-making methods; determination of indicator weights; contextual drivers; description and scoring of indicators; multi-criteria decision analysis methods for ranking alternatives; and system simulation parameter values, ranges, and distributions (PDF)
