# Supplementary material for: DMsan: A Multi-Criteria Decision Analysis Framework and Package to Characterize Contextualized Sustainability of Sanitation and Resource Recovery Technologies
Source: ACS Environ Au. 2023 Mar 27;3(3):179–92. doi: 10.1021/acsenvironau.2c00067 (PMC10197171; doi:10.1021/acsenvironau.2c00067)
Supplement: Supplementary file 1 — vg2c00067_si_001.pdf [file vg2c00067_si_001.pdf]

Supporting Information for

DMsan: A multi-criteria decision analysis framework and package to characterize contextualized sustainability of sanitation and resource recovery technologies

Hannah A.C. Lohman,[1,†] Victoria L. Morgan,[2,†,§] Yalin Li,[2,3] Xinyi Zhang,[1] Lewis S. Rowles,[2,¶] Sherri M. Cook,[4] Jeremy S. Guest[1,2,3,*]

Author Affiliations:

[1] Department of Civil and Environmental Engineering, 3221 Newmark Civil Engineering Laboratory, University of Illinois Urbana-Champaign, 205 N. Mathews Avenue, Urbana, IL 61801, USA

[2] Institute for Sustainability, Energy, and Environment, University of Illinois Urbana-Champaign, 1101 W. Peabody Drive, Urbana, IL 61801, USA

[3] DOE Center for Advanced Bioenergy and Bioproducts Innovation, University of Illinois Urbana-Champaign, 1206 W. Gregory Drive, Urbana, IL 61801, USA

[4] Department of Civil, Environmental, and Architectural Engineering, University of Colorado Boulder, 1111 Engineering Drive, Boulder, CO 80309, USA

[†] H.A.C. Lohman and V.L. Morgan contributed equally to this work.

[§] Present address: Hazen and Sawyer, 2420 Lakemont Avenue, Suite 325 Orlando, FL 32814, USA

[¶] Present address: Department of Civil Engineering and Construction, Georgia Southern University, 201 COBA Drive, BLDG 232 Statesboro, GA 30458, USA

*To whom correspondence should be addressed: jsguest@illinois.edu; 217-244-9247

Table of Contents (40 total pages)

S1. Literature review of decision-making methods for selection of sanitation systems..... 4

S2. Determination of indicator scores 6

S2.1. Description and scoring of technical indicators 7
S2.1.1. Resiliency and robustness sub-criterion indicators 8
S2.1.2. Feasibility sub-criterion indicators 9
S2.1.3. Flexibility sub-criterion indicators..... 13

S2.2. Description and scoring of resource recovery indicators..... 14
S2.2.1. Resource recovery feasibility sub-criterion indicators 14

S2.3. Description and scoring of environmental indicators 16
S2.3.1. Life cycle environmental impacts sub-criterion indicators 16

S2.4. Description and scoring of economic indicators 17
S2.4.1. Net costs sub-criterion indicators 18

S2.5. Description and scoring of social indicators..... 18
S2.5.1. Job creation sub-criterion indicators..... 19
S2.5.2. End-user acceptability sub-criterion indicators..... 20
S2.5.3. Property manager acceptability sub-criterion indicators..... 22

S3. Determination of indicator weights 23

S3.1. Technical indicator contextual drivers..... 23
S3.1.1. Resiliency and robustness contextual drivers 23
S3.1.2. Feasibility contextual drivers 23
S3.1.3. Flexibility contextual drivers..... 24

S3.2. Resource recovery indicator contextual drivers 25

S3.3. Environmental and economic indicator contextual drivers 25

S3.4. Social indicator contextual drivers..... 25
S3.4.1. Job creation contextual drivers..... 25
S3.4.2. End-user acceptability contextual drivers 26
S3.4.3. Property manager acceptability contextual drivers..... 27

S3.5. The analytical hierarchy process (AHP) for calculating indicator weights..... 27

S4. Multi-criteria decision analysis methods to evaluate sanitation alternatives 29

S4.1. Criteria weight scenarios..... 29

S4.2. The technique for order by preference of similarity (TOPSIS) method for calculating performance score and rank of sanitation alternatives 30

S5. System simulation with techno-economic analysis and life cycle assessment 31

Table S1. Terminology use to inform and describe the DMsan package.

Term	Acronym	Description
Decision-making for sanitation and resource recovery systems	DMsan	A Python package for decision-making of sustainable sanitation and resource recovery systems in low-income settings.
Multi-criteria decision analysis	MCDA	A methodology that compares multiple conflicting criteria and preferences to measure the probability that an alternative will outrank other alternatives.
Analytical hierarchy process	AHP	A method of MCDA that uses pairwise comparisons to generate criteria weights and/or rankings of alternatives.
Technique for order by preference of similarity	TOPSIS	A method of MCDA that uses Euclidian distances from the best option to the worst option to generate rankings of alternatives.
Life cycle assessment	LCA	A technique to quantify the environmental impacts of a technology.
Techno-economic analysis	TEA	A technique to quantify the economic costs of a technology.
Alternative	-	Within MCDA, an option that is compared against other options (i.e., Alternative A, Alternative B, and Alternative C).
Criterion	-	The main or principal decision-making categories of the framework (i.e., technical, environmental, resource recovery, economic, and social).
Criterion weight	-	The weight given to a criterion to represent its significance in MCDA.
Sub-criterion	-	The set of decision-making categories that fall under the criterion
Indicator	-	A specific aspect in which the technology alternative is assessed (e.g., number of jobs created).
Indicator contextual driver	-	Used to generate an indicator weight (e.g., extent of training, community preference, population growth).
Indicator weight	-	The contextual/location importance for a specific indicator and is generated using indicator contextual driver through AHP.
Indicator score	-	The value assigned to an indicator to measure the ability of an individual aspect of a technology before normalization and weighting.
Performance score	-	The total quantitative score generated for each alternative and is used for ranking.

S1. Literature review of decision-making methods for selection of sanitation systems

A Scopus search using title, abstract, and keyword search terms was conducted to explore the factors involved in sanitation decision-making for low-income communities. Search terms accounted for a variety of terms related to sanitation, decision-making, resource-limited settings, developing economies, and system selection (**Table S2**). The search was limited to articles published from 1960 to September 2020.

Table S2. Literature review search terms. These terms were used in a Scopus search to identify peer-reviewed publications related to decision-making of sanitation systems in low-income communities.

Category	Included search terms	Excluded search terms
Sanitation	Sanitation, wastewater, household sanitation	Covid, Industr*, meat*
Decision-making	Decision*, multi-criteria*, multicriteria*	Covid, Industr*, meat*
Resource-limited settings	Marginalized, low-income, "low income", poverty*, trib*, indigenous*, resource-limited, "resource limited", underprivileged, underserved, deprived, disadvantaged, impoverished, vulnerable, native*, peri-urban, rural, slum, "off the grid", off-the-grid	Covid, Industr*, meat
Developing economies	All countries identified as a "developing economy" by World Economic Situation and Prospects in 2019[1]	Covid, Industr*, meat
System selection	Tech*, system*, selection	Covid, Industr*, meat

A literature review was conducted to identify the commonly used criteria and indicators in sanitation decision-making studies. Four main criteria were identified: environmental (32 out of 35 papers), economic (29 out of 35 papers), technical (28 out of 35 papers), and social (26 out of 35 papers). The most used indicators (appearing in 3 or more papers) are summarized below (**Table S3**), with all indicators used across published literature appearing in the later sections describing criteria indicators and scoring.

Table S3. Summary of criteria and sub-criteria/indicators found in decision-making papers focused on sustainable sanitation system selection.

Criteria Type	Frequency	Sub-criterion/Indicator	Frequency
Environmental	32	Energy consumption	12
		Air contamination	10
		Sludge production	5
		Water contamination	4
		Water consumption	4
		Odor	4
		Eutrophication	4
Economic	29	Operation and maintenance costs	14
		Capital costs	13
		Resource recovery, profit values	5
Technical	28	Reliability or robustness or resiliency	12
		Operation and maintenance requirement, manpower, complexity, feasibility	11
		Land requirement	5
		Flexibility, adaptability	4
Social	26	Socio-cultural acceptability	10
		Job creation	3
		Policy and government compatibility	5

Table S4. Summary of decision-making techniques and MCDA methodologies found in decision-making papers focused on sustainable sanitation system selection.

Paper	Decision-support technique	MCDA methodology	Reference
Yoon et al., 2019	MCDA	Choosing by advantage	[2]
Katukiza et al., 2010	MCDA	AHP	[3]
Awad et al., 2019	LCA	-	[4]
Kalbar et al., 2016	MCDA and LCA	TOPSIS	[5]
Kalbar et al., 2013	LCA	-	[6]
Khattiyavong and Lee, 2019	MCDA	TOPSIS	[7]
Lizot et al., 2020	MCDA	AHP and ELECTRE	[8]
Kalbar et al., 2012	MCDA and LCA	TOPSIS	[9]
Kamble et al., 2017	MCDA and LCA	AHP and ELECTRE	[10]
Kalbar et al., 2012	MCDA and LCA	TOPSIS	[11]
Padron-Paez, 2020	MCDA	TOPSIS	[12]
Zhang et al., 2020	MCDA	AHP	[13]
Promentilla et al., 2018	MCDA	AHP	[14]
Singhirunnusorn & Stenstrom, 2009	MCDA	ELECTRE	[15]
Vidal et al., 2019	MCDA	ELECTRE	[16]
Livia et al., 2020	MCDA	AHP	[17]
Sadr et al., 2018	MCDA	AHP	[18]
Salisbury et al., 2018	MCDA	Multi-attribute utility theory	[19]
Anaokar et al., 2018	MCDA	TOPSIS	[20]
Mucha et al., 2016	MCDA	TOPSIS	[21]
McConville et al., 2020	MCDA	Qualitative	[22]
Perez et al., 2010	MCDA	AHP	[23]
Seleman & Bhat, 2016	MCDA	AHP	[24]
Willetts et al., 2013	MCDA	Other/not mentioned	[25]
Gao et al., 2017	LCA	-	[26]
Singh et al., 2017	Cost benefit analysis	-	[27]
Hashemi and Boudaghpour, 2020	Cost benefit analysis	-	[28]
Kerstens et al., 2015	Cost benefit analysis	-	[29]
Retamal et al., 2011	Cost benefit analysis	-	[30]
Spuhler, 2018	Appropriateness assessment	-	[31]
Spuhler, 2020	Appropriateness assessment	-	[32]
Simiyu, 2017	Strengths, weaknesses, opportunities, threats (SWOT) analysis	-	[33]
Triantafyllidis et al., 2018	Agent-based modeling	-	[34]
Malekpour et al., 2013	Probabilistic modeling	-	[35]
Abdel Wahaab et al., 2020	Decision support systems	-	[36]

S2. Determination of indicator scores

Five criteria, consisting of technical, resource recovery, environmental, economic, and social, are used within DMsan to evaluate the performance of sanitation systems. This section provides details about the indicators and how to calculate or assign indicator scores.

Indicator scores are assigned for alternatives A, B, and C in each indicator scoring table in this section. System details are described in the main text of the manuscript and in Trimmer et al.[43] In summary, Alternative A is the existing sanitation system incorporating pit latrines, vacuum collection trucks, centralized treatment (sedimentation, solids drying beds, and lagoons), and recovery of nutrients for fertilizer (dried solids and nutrient-rich liquid effluent). Alternative B replaces the existing centralized treatment with an anaerobic baffled reactor, solids drying beds, and an additional planted bed for liquid treatment with solid and liquid nutrients recovered for land application and biogas for cooking fuel. Alternative C replaces the existing pit latrines with container-based urine-diverting dry toilets that use urine handcart and solids truck transportation to bring resources to the centralized treatment facility described in Alternative A, excluding sedimentation, as liquids and solids are already separated. Alternative C increases the nutrient recovery potential (relative to Alternatives A and B) through source separation.

Table S5. Summary of criteria, sub-criteria, and indicators included in DMsan. Qualitative indicator scores are manually assigned using the predefined ranges described in this section (**Section S2**), and quantitative indicator scores are simulated using QSDsan (**Section S5**). Simulated indicator scores and calculations were informed using the methods and calculations described in Trimmer et al.[43] Indicators with an asterisk were excluded in the Uganda example highlighting the use of DMsan because property managers were not responsible for system disposal and cleaning efforts.

Criteria	Sub-criteria	Indicator	Type of Input	Table in SI
Technical	Resiliency & robustness	User interface robustness	Manual score input	Table S7
		Resiliency of treatment type	Manual score input	Table S8
	Feasibility	Accessibility to parts	Manual score input	Table S9
		Transportation feasibility	Manual score input	Table S10
		Construction skills required	Manual score input	Table S11
		Operation and maintenance skills required	Manual score input	Table S12
	Flexibility	Population flexibility	Manual score input	Table S13
		Power outage flexibility	Manual score input	Table S14
		Drought flexibility	Manual score input	Table S15
Resource recovery	Resource recovery feasibility	Water recovery	Simulated	-
		Nitrogen recovery	Simulated	Table S16
		Phosphorous recovery	Simulated	Table S16
		Potassium recovery	Simulated	Table S16
		Energy recovery	Simulated	Table S17
		Supply chain feasibility	Manual score input	Table S18
Environmental	Life cycle environmental impacts	Damage to ecosystems	Simulated	Table S20
		Damage to human health	Simulated	Table S20
		Damage to resource availability	Simulated	Table S20
Economic	Net costs	Annual cost per capita	Simulated	Table S22
Social	Job creation	Total jobs created	Simulated	Table S24
		High-paying jobs created	Simulated	Table S24
	End-user acceptability	Disposal frequency	Simulated	Table S25
		Cleaning requirement	Manual score input	Table S26
		Privacy	Simulated	Table S27
		Odor and flies	Manual score input	Table S28
		Security	Manual score input	-
	Property manager acceptability	Disposal frequency*	Manual score input	-
		Cleaning requirement*	Manual score input	-

S2.1. Description and scoring of technical indicators

The technical criteria category encompasses the aspects of how a system or technology performs and functions. Indicators identified through the literature review for this criterion included reliability,[5,8,10,13,15,17,32,36,44] resiliency,[32] robustness,[3,9,16,19,22,25,32] flexibility,[5,10,36,44] adaptability,[3,18,32,34] durability,[5,10,19] feasibility,[25,32,44] operation and maintenance requirement,[3,5,9,10,15,27,31,35,36,44] and other indicators (**Table S6**). Using the results of the literature review, the sub-criteria selected for DMsan include resilience, feasibility, and flexibility.

Table S6. Technical indicators identified in the literature review.

Technical Indicator	Reference(s)
O&M/manpower requirement	3,9,10,15,27,31,35,36,44
Spare parts	31
Feasibility/ Applicability	25,32,44
Reliability	5,8,10,13,15,17,32,35,36,44
Adaptability	3,18,32,34
Resilience	32
Robustness	3,9,16,19,22,25,32
Compatibility with existing infrastructure	2,24,32
Resource recovery	12,15–17,19,24,27,29,32,36,44
Treatment objectives	13,15–17,19,29
Complexity	8,17,18,25,36
Continuity of facility provision and operation	15
Long term system performance	15
Short term system performance	15
Ease of construction, installation, and startup	15,18
Flexibility and modularity	5,10,36,44
Consumables consumption	36
New technology	2
Expansion feasibility	2
Level of outside monitoring	19
Durability	5,10,19
% Yield	27
Ease of operation	21
Availability of material locally	24
Local skills	24
Replicability	5,8,10
Simulation of sustainable behavior	5
Risk of plan not being completed	25
Ability to obtain materials	3
Coordination with local climate	44
Coordination with local facilities	44
Land Requirement	10
Temperature	20,31
Flooding	31
Vehicular access	31
Slope	31
Soil type	31
Groundwater depth	27,31
Excavation	31
Land use	5,7,9,13,15,17,18,21,27,29,32,36
Compatibility with ecological zones	32
Transport distance	36
Water supply reliability	35

S2.1.1. Resiliency and robustness sub-criterion indicators

The resiliency and robustness sub-criterion is focused on the ability of a system to achieve adequate performance under a variety of conditions for long periods of time. Some factors that will affect resiliency and robustness include adaptability to inputs at the user interface and the type of treatment (e.g., biological, chemical, etc.).

User interface robustness (T1). The user interface describes the type of toilet, pedestal, pan, or urinal that the user interacts with to access the sanitation system. There are five categorized types of interfaces: dry toilet, pour-flush toilet, cistern flush toilet, urine-diverting dry toilet (UDDT), and urine-diverting flush toilet (UDFT). Successful user interface selection is influenced by the following factors: availability of water for flushing, habits and preferences of the users, special needs of user groups, local availability of materials, and compatibility with the collection and storage/treatment or conveyance of the system.[45] To identify the complexity in using these interfaces and on-site storage, a 1 to 5 scale was created where one is the least complex and most simple to use and five is the most complex and most difficult to use according to former studies (**Table S7**).[45] The scale below was used to give a user interface robustness score to each alternative. The less complex the user interface, the less likely it will lead to user failure (if there isn't adequate training in place). Alternatives A and B have a pour flush toilet and score of 4, and Alternative C has a UDDT with a score of 2.

Table S7. Indicator score descriptions to assess user interface robustness and their application to Alternatives A - C.

Indicator Score	Description	Example	Alternative A	Alternative B	Alternative C
1	Highly complex user interface that has labor-intensive maintenance, requires training and acceptance to be used correctly, is prone to misuse and clogging, and requires a constant source of water.	Urine-diverting flush toilet (UDFT)			
2	Complex user interface that requires training and acceptance to be used correctly, is prone to misuse and clogging with feces, and the excreta pile is visible.	Urine-diverting Dry Toilet (UDDT)			X
3	Moderately complex user interface that may not be repaired locally, requires a constant source of water, and operating costs depend on the price of water.	Cistern Flush Toilet			
4	Simple user interface that requires a constant source of water and coarse dry cleansing materials may clog the water seal.	Pour Flush Toilet	X	X	
5	Most simple user interface that does not requires training, not prone to clogging, and does not require water use but attracts flies and odor.	Dry toilet			

Resiliency of treatment type (T2). Treatment type resiliency is based on the type of treatment employed for wastewater treatment: anaerobic (e.g., anaerobic lagoons, anaerobic reactors, etc.), aerobic (e.g., activated sludge, fixed-bed and moving bed bioreactors, aerobic membrane bioreactors, biological trickling filters, etc.), and chemical/thermal (e.g., disinfection, ion-exchange, gasification, incineration, pyrolysis, etc.). We assumed that anaerobic systems have low resilience due an increased risk in fouling and require more operation and maintenance,[46] aerobic systems have moderate resilience, and thermal or chemical will have high resilience. Decision-makers and technology developers interested in evaluating systems outside of conventional wastewater treatment (e.g., novel technologies and resource recovery alternatives) can modify the scoring scale to evaluate the relative difference in resilience across systems and apply scores accordingly. An indicator scale of 1 to 3 was created, where one is low resilience and three is high resilience (**Table S8**). Each alternative in the illustration used anaerobic treatment and received a score of 1.

Table S8. Indicator score descriptions to assess resiliency of treatment type and their application to Alternatives A - C.

Indicator Score	Description	Example	Alternative A	Alternative B	Alternative C
1	Low resilience for anaerobic systems that are likely to need more maintenance and have a higher risk of fouling.	Anaerobic lagoons, anaerobic reactors	X	X	X
2	Moderate resilience for aerobic systems as they require maintenance to keep the microorganisms stabilized.	Activated sludge, fixed bed and moving bed bioreactors, aerobic membrane reactors, trickling filters			
3	High resilience for chemical/thermal treatment systems.	Disinfection, ion-exchange, gasification, incineration			

S2.1.2. Feasibility sub-criterion indicators

The feasibility sub-criterion is related to the availability of required resources for a system to operate. In the literature review, feasibility, adaptability, applicability, and complexity were used to assess if a community had the resources (e.g., land, human resources, parts, etc.) needed to maintain the system. The MCDA indicators to quantify the feasibility of the system are accessibility to parts in case a part needs to be replaced, transportation feasibility related to the complexity of conveyance, construction skills required to build the system, and operation and maintenance (O&M) expertise needed for the system to function.

Accessibility to parts (T3). Sanitation systems often fail within a couple years of implementation, often due to systems breaking without attention to how they can be repaired. The accessibility to parts indicator focuses on how easily parts can be obtained during the construction and maintenance phases of an alternative's lifetime. A qualitative score of one to five is used where one represents parts that have very low accessibility (e.g., custom electrodes) and five represents parts that have very high accessibility (e.g., concrete) (**Table S9**). In this example, Alternatives A and C are assumed to have very high accessibility (indicator score = 5), and Alternative B has high accessibility (indicator score = 4) because its biogas bioreactor may have some parts that need to be shipped.

Table S9. Indicator score descriptions to assess accessibility to parts and their application to Alternatives A - C.

Indicator Score	Description	Example	Alternative A	Alternative B	Alternative C
1	Very low accessibility due to the requirement of custom parts (e.g., parts produced by a single entity such as those produced for novel systems using proprietary information).	Custom electrodes			
2	Low accessibility due to parts required for advanced treatment of wastewater (i.e., any process that reduces impurities in wastewater below what is attainable through conventional secondary or biological treatment).	Parts for tertiary filtration and disinfection			
3	Moderate accessibility that requires the shipment of majority of parts.	Trickling filter			
4	High accessibility as most parts may be locally available or easily accessible but some parts may need to be shipped.	Pour-flush toilet, settler, biogas reactor		X	
5	Very high accessibility as parts only require plastic, concrete, and other easily accessible or local material.	Dry toilet, pit latrine, sedimentation tank	X		X

Transportation feasibility (T4). The transportation feasibility indicator evaluates the complexity of the conveyance system required for each alternative. Transportation feasibility is scored on a scale of 1 to 7, where 7 is low complexity and 1 is the highest complexity (**Table S10**). In this illustration, Alternatives A and C use tanker trucks (indicator score = 5), and Alternative C uses trucks and pushcart operators (indicator score = 6). For alternatives that have the necessary conveyance infrastructure already in place, DMsan users can select score 7 to represent no additional infrastructure required. For systems without conveyance of waste (e.g., scenarios in which a new pit latrine is dug instead of pumped to empty), DMsan users can set the weight of the indicator to 0 indicate that it is not being included.

Table S10. Indicator score descriptions to assess transportation feasibility and their application to Alternatives A - C.

Indicator Score	Description	Example	Alternative A	Alternative B	Alternative C
1	Most complex transportation infrastructure required.	Transfer station			
2	Very high complexity of transportation infrastructure required.	Conventional gravity sewer			
3	High complexity of transportation infrastructure required.	Solids-free sewer			
4	Moderate complexity of transportation infrastructure required.	Simplified sewer			
5	Low complexity of transportation infrastructure required.	Motorized emptying and transport (tanker trucks)	X	X	
6	Very low complexity of transportation infrastructure required.	Human-powered emptying and transport (hand carts)			X
7	Most simple transportation infrastructure required / appropriate infrastructure already in community.	Jerrycan or tank			

Construction skills required (T5). The construction skills required can be used to evaluate the complexity of implementation. The indicator is scored from 1 to 5, where one is very advanced skills required and five is very minimal skills required to build the system (**Table S11**). The *Compendium of Sanitation Technologies and Systems*[45] provides insight into the construction complexity required for conventional technologies, such as constructed wetlands, biogas reactors, and other components. The scores and descriptions were informed by the construction complexities described in the compendium; however, DMsan users can assign construction skill levels through conversations with technology developers or using their own judgement (following the examples in the table) if engineers/technology developers are not available for feedback during the decision-making phase. Alternative A does not require any construction as the system is already existing and receives a score of 5. Alternative B does not require any construction for storage but does require the construction of an anaerobic baffled reactor, unplanted and planted drying beds (solids), and planted bed (liquids) and is given a score of 2. Alternative C does not require construction as the solids and liquids are transported to the site to be treated by the existing treatment system, so it is given a score of 5.

Table S11. Indicator score descriptions to assess construction skills required and their application to Alternatives A - C.

Indicator Score	Description	Example	Alternative A	Alternative B	Alternative C
1	Very advanced construction skills required.	Advanced electronics, specialty welding			
2	Advanced construction skills required.	Constructed wetlands and reactor fabrication		X	
3	Moderate construction skills required.	System assembly			
4	Some construction skills required.	Connecting systems to utilities, bolt-on attachment			
5	Minimal to no construction skills required.	On-site treatment shipped in a container, existing system	X		X

Operation and maintenance skills required (T6). The final feasibility indicator is related to the operation and maintenance skills required for the system. This indicator is measured on a scale of 1 to 5, where a score of one requires very advanced operation and maintenance skills and five requires the simplest operation and maintenance skills (**Table S12**). The scores and descriptions were informed by the operation and maintenance complexities described in the compendium; however, DMsan users can assign construction skill levels through conversations with technology developers or using their own judgement (following the examples in the table) if engineers/technology developers are not available for feedback during the decision-making phase. For Alternative A, a score of 3 (moderate O&M) was assigned because the centralized treatment involves constructed wetlands and sedimentation. Alternative B uses an anaerobic baffled reactor, which resulted in a score of 2 (advanced O&M). Alternative C is the same as Alternative A when it comes to operation and maintenance required for treatment, so the score for this alternative is also 3 (moderate O&M).

Table S12. Indicator score descriptions to assess operation and maintenance skills required and their application to Alternatives A - C.

Indicator Score	Description	Example	Alternative A	Alternative B	Alternative C
1	Very advanced operation and maintenance skills required.	Chemical treatment			
2	Advanced operation and maintenance skills required.	Anaerobic sludge basin, anaerobic baffled reactor		X	
3	Moderate operation and maintenance skills required.	Constructed wetlands, sedimentation	X		X
4	Some operation and maintenance skills required.	Drying beds, lined pit latrines			
5	Minimal to no operation and maintenance skills required.	Fill and Cover/Arborloo, Dehydration vaults, Composting chambers			

S2.1.3. Flexibility sub-criterion indicators

The final sub-criterion of the technical criterion is related to the flexibility of a sanitation system to withstand societal and environmental changes while maintaining ability. The flexibility of a system is characterized by flexibility to population growth, electricity blackouts, and drought.

Population flexibility (T7). Population flexibility relates to the ability of a sanitation system to withstand population changes, specifically population growth. It is quantified using a scale of 1 to 3, where 1 is assigned to a system with low flexibility (handles less than a 10% increase in population), 2 has some flexibility to population growth (handles a 10-25% increase in population), and 3 has the most flexibility (handles greater than a 25% increase in population) (**Table S13**). Alternatives A, B, and C can handle a 10-25% population increase and were assigned a score of 2. Population increases for Alternatives A, B, and C were assigned using the author's expert judgement. DMsan users can work with technology developers or use their own expert judgement to evaluate the population flexibility of alternatives.

Table S13. Indicator score descriptions to assess population flexibility and their application to Alternatives A - C.

Indicator Score	Description	Alternative A	Alternative B	Alternative C
1	Can handle less than a 10% increase in population.			
2	Can handle a 10-25% increase in population.	X	X	X
3	Can handle more than a 25% increase in population.			

Power outage flexibility (T8). Power outage flexibility is related to a system's energy requirement and its ability to operate without a constant energy source. It is characterized on a scale of 1 to 3, where 1 always requires grid electricity to run as designed, 2 can handle blackouts of up to 24 hours, and 3 is no dependence on grid electricity (**Table S14**). The indicator score is assigned based on the least flexible unit process of the system (e.g., if one step of the sanitation service chain always requires electricity and the remaining steps do not require electricity, the system will receive a score of 1). All Alternatives always require grid electricity to run as designed and do not have any onsite storage or generation of electricity, so all alternatives were assigned a score of 1.

Table S14. Indicator score descriptions to assess power outage flexibility and their application to Alternatives A - C.

Indicator Score	Description	Example	Alternative A	Alternative B	Alternative C
1	Always requires grid electricity to run as designed.	No onsite storage or generation of electricity	X	X	X
2	Can handle blackouts of up to 24 hr.	Onsite storage or generation of electricity capable of supporting electrical demands for 24 hours.			
3	No dependence on grid electricity.	No grid electricity requirements; onsite storage or generation of electricity capable of supporting the entire system			

Drought flexibility (T9). The ability of a sanitation system to withstand drought due to its reliance on water is measured on a scale of 1 to 3, where is 1 highly dependent on water (always requires water – cleaning/maintenance, treatment, and conveyance), 2 some dependence on water (only sometimes required water – cleaning/maintenance only), and 3 is no dependence on water (never needs water – produces enough water onsite through water recovery) (**Table S15**). Alternatives A and B both consider an existing sewer network that is fed by flush toilets (highly dependent on water) and receive scores of 1. Alternative C only requires water for cleaning of the urine-diverting dry toilets (some dependence on water) and receives a score of 2.

Table S15. Indicator score descriptions to assess drought flexibility and their application to Alternatives A - C.

Indicator Score	Description	Example	Alternative A	Alternative B	Alternative C
1	Highly dependent on water.	Flush toilets	X	X	
2	Some dependence on water.	Water only required for cleaning or maintenance			X
3	No dependence on water.	Water never required			

S2.2. Description and scoring of resource recovery indicators

Resource recovery is important to consider in contexts with low resource access (e.g., water, energy, nutrients) and regions shifting to circular economics with more sustainable resource acquisition. Six indicators were selected for this criterion to quantify water, energy, and nutrient recovery from the system and the complexity of the supply chain necessary to deliver recovered resources.

S2.2.1. Resource recovery feasibility sub-criterion indicators

Water recovery (RR1). Water recovery is calculated as the volume (e.g., L per day) of water recovered to be reused within the system (e.g., flush water) or used for purposes outside of the system boundary (e.g., irrigation, household cleaning, etc.). This indicator was excluded from this analysis because all three systems did not have water recovery in their designs; however, the indicator should be included in future analyses of systems that include water recovery.

Nitrogen, phosphorous, and potassium recovery (RR2, RR3, RR4). The second, third, and fourth indicators quantify the nutrient recovery from the system. Recovered nitrogen, phosphorus, and potassium are calculated in QSDsan using an expected user's dietary intake (i.e., daily calories, vegetal protein, animal protein), expected food waste, and nutrient losses along the system (e.g., ammonia volatilization) (**Table S16**).[43] The calculations and simulations are further discussed in **Section S5**.

Table S16. Simulated nutrient recovery indicator scores: baseline [5th percentile, 95th percentile].

Indicator	Unit	Alternative A	Alternative B	Alternative C
Nitrogen (N) recovery	% N recovered	10 [3, 14]	34 [14, 40]	78 [67, 82]
Phosphorus (P) recovery	% P recovered	41 [30, 53]	79 [61, 93]	56 [48, 67]
Potassium (K) recovery	% K recovered	76 [66, 83]	76 [66, 83]	96 [92, 97]

Energy recovery (RR5). The fifth indicator quantifies the energy recovered from the system as a fraction of the COD input recovered as energy (**Table S17**). Alternatives A and C do not have energy recovery, and the energy recovered in biogas for Alternative B was simulated (detailed in **Section S5**).

Table S17. Simulated energy recovery scores: baseline [5th percentile, 95th percentile].

Indicator	Unit	Alternative A	Alternative B	Alternative C
Energy recovery	% COD recovered for energy	0	39 [23, 47]	0

Supply chain feasibility (RR6). The sixth indicator to characterize the resource recovery criterion is related to the complexity and feasibility of the supply chain to deliver the resources. For inefficient supply chains (based on the contextual driver of the country), less complexity in steps (e.g., on-site reuse) to distribute resources is preferred. A score of 1 to 4 is assigned based on the complexity to deliver the recovered resources to a consumer, where recovered water onsite for flush water scores a one, biogas recovered onsite for a wastewater treatment plant scores a 2, nutrients recovered for agriculture purposes scores a three, and biogas for offsite distribution scores a four (**Table S18**). Alternatives A and C produces recovered liquid as nutrient-rich water for irrigation and recovered solids as fertilizer, which results in a score of 3. Alternative B produces biogas for cooking fuel and distributes recovered solids and liquids, which is more difficult and given a 4.

Table S18. Indicator score descriptions to assess supply chain feasibility and their application to Alternatives A - C.

Indicator Score	Description	Example	Alternative A	Alternative B	Alternative C
1	Recovered water/liquids for onsite use	Treatment of liquids that can be directly pumped back into the system for flush water			
2	Recovered biogas/energy for onsite applications	Biogas is onsite at the facility (i.e., to cover energy for the treatment plant)			
3	Recovered solid/liquid nutrients for agricultural applications	Nutrients from liquids and solids are recovered that will be distributed to agriculture	X		X
4	Recovered biogas/energy for offsite applications	Biogas is distributed offsite to business and/or homes		X	

S2.3. Description and scoring of environmental indicators

The environmental criterion encompasses all aspects of how a technology impacts the environment. Articles in the literature review most often included energy and water consumption, air and water contamination, and sludge production as environmental indicators (**Table S19**).

Table S19. Environmental indicators identified in the literature review.

Environmental Indicator	Reference(s)
Water consumption	[19,24,31,32]
Energy consumption	[6,7,12,16,18,19,25–27,29,31,32,36]
Water contamination	[6,24,32,35]
Air contamination (GHG, CO_2, GWP, etc.)	[4–7,9,12,16,25,26,32,34]
Beneficial landscape integration	[32]
Sludge production	[4,7,8,13,17,36]
Odor	[13,17,22,36,44]
Noise	[13,17,36]
Acidification	[4,6]
Eutrophication	[4–6,9,10]
Toxicity	[6]
Sustainability	[9]
Environmental impact	[2,18,21,44]
Abiotic depletion	[4]
Ozone layer depletion	[4]
Fresh water aquatic ecotoxicity	[4]
Terrestrial ecotoxicity	[4]
Photochemical oxidation	[4]
Water quality	[18,25]
Impact on forest	[24]
Ability to adapt to climate change	[25]
Nutrient reuse	[25]
Environmental pollution	[3]
Exposure to pathogens	[3]
Reach treatment requirement	[44]
Risk	[44]
Global Warming Potential	[8,10]
Resource Recovery	[10]
Organic Matter Efficiency	[8]
Waste	[34]

S2.3.1. Life cycle environmental impacts sub-criterion indicators

Life cycle assessment is used to calculate the environmental indicators. The ReCiPe life cycle assessment methodology was selected because it is used globally, and the endpoint indicators can be compared with universal units (points).[47] The indicators include damage to the ecosystem quality, damage to human health, and damage to resource availability and were calculated using the hierarchist cultural perspective (other perspectives include individualist and egalitarian) because it is considered to be the default model (**Table S20**).[47] Negative results indicate the damage offsets due to resource recovery are greater than the damage produced by the system itself (i.e., the lower the damage score, the better performance). The calculations and simulations are further discussed in **Section S5**.

Damage to ecosystem quality (Env1). This endpoint indicator accounts for the local relative species loss in terrestrial, freshwater and marine ecosystems over space and time.[47] Damage pathways for this indicator include damage to freshwater, terrestrial, and marine species as a result of global warming, water use, freshwater ecotoxicity, freshwater eutrophication, tropical ozone, terrestrial ecotoxicity, terrestrial acidification, land use or transformation, and marine ecotoxicity.

Damage to human health (Env2). This endpoint indicator is quantified as disability adjusted life years (DALYs), and represents the years lost for a person due to a disease or accident.[47] DALYs were converted to points to allow comparison across environmental indicators. Damage pathways for this indicator include increase in respiratory disease, increase in various types of cancer, increase in other diseases or causes, and increase in malnutrition due to particulate matter, topical ozone formation, ionizing radiation, stratospheric ozone depletion, human toxicity (cancer and non-cancer), global warming, and water use.

Damage to resource availability (Env3). This final endpoint indicator is quantified as the extra costs involved for future mineral and fossil resource extraction converted to points.[47] The increased extraction costs are a consequence of mineral and fossil resources used.

Table S20. Simulated life cycle assessment indicator scores: baseline [5th percentile; 95th percentile].

Indicator	Unit	Alternative A	Alternative B	Alternative C
Damage to ecosystems	points•capita^{-1}•year^{-1}	-1,599<br>[-4,872; -1,297]	-3,643<br>[-10,410; -2,868]	-6,111<br>[-23,570; -4,660]
Damage to human health	points•capita^{-1}•year^{-1}	25.8<br>[12.2; 44.0]	11.4<br>[4.8; 23.1]	7.0<br>[3.2; 14.9]
Damage to resources	points•capita^{-1}•year^{-1}	0.02<br>[-0.10; 0.47]	-0.47<br>[-0.69; 0.12]	0.50<br>[-0.19; 3.50]

S2.4. Description and scoring of economic indicators

Economic feasibility is a critical factor in driving decision-making. Common indicators in literature include capital cost, operation and maintenance cost and recovered resource value (**Table S21**). Net annualized cost was selected as the primary indicator for the economic criterion to include the common cost indicators in literature.

Table S21. Economic indicators identified in the literature review.

Economic Indicator	Reference(s)
Capital/investment cost	2,3,8,9,13,15–19,21,22,25,27,28,32,34,36,44
Capital replacement	11
Residual value	11
Electricity cost	11
Operation and maintenance costs	2–4,8,13,15–19,21,22,24,27,32,35,36,44
Total cost	4,12
Recovered resource value	3,4,22,25,32
Life cycle cost	8–10,29
Profit	27,36
Impact on property value	2
Collected tax	2
Tax benefits	2
Amortization cost	4
Net cost	4
Revenue	11,27
Internal rate of return	27
Payback period	27
Combined yield	27
Willingness to pay	24
Ability to pay for improvement	24
Net present worth	5,25,30
Cost Sharing	25
Land	3,8,25,44
Levelized Costs	30
Energy - biogas recovery	3
Disposal	28,44
Construction Cost	28
Manpower	8

S2.4.1. Net costs sub-criterion indicators

Techno-economic analysis was used to calculate the net annualized cost for each alternative. The calculation includes costs related to construction (capital), operation and maintenance, labor, transportation, consumables, electricity, and all expenses required to construct and maintain each system.[43] The calculations and simulations are further discussed in **Section S5** with complete details and modeling assumptions in Trimmer et al.[43]

Annual cost per capita (Econ1). The net annualized cost is normalized per capita and year to determine an annual cost per capita. The techno-economic analysis is conducted assuming Alternative A serves 456,667 users (40,000 on existing sewer and 416,667 on latrines) and has an 8-year lifetime, Alternative B serves 50,000 users and has a 10-year lifetime, and Alternative C serves 456,667 users (40,000 on existing sewer and 416,667 on latrines) and has an 8-year lifetime. Using these assumptions, Alternative A has an annual cost per capita of 14.23 USD·capita^{-1}·year^{-1}, Alternative B has an annual cost per capita of 7.34 USD•capita^{-1}•year^{-1}, and Alternative C has an annual cost per capita of 22.06 USD•capita^{-1}•year^{-1} (**Table S22**).

Table S22. Simulated annual cost per capita indicator scores: baseline [5th percentile, 95th percentile].

Indicator	Unit	Alternative A	Alternative B	Alternative C
Annual cost per capita	USD·capita^{-1}•year^{-1}	14.23 [10.76, 23.04]	7.34 [4.25, 15.88]	22.06 [16.60, 30.29]

S2.5. Description and scoring of social indicators

The final criterion selected for the package focused on social drivers of decision-making. Often overlooked, the criterion is critical in understanding how a technology will be socially sustainable in a specific location. In the review, researchers considered job creation, socio-cultural acceptability, compatibility with policy, visual impact, and additional indicators (**Table S23**). As a result, within the DMsan package, the social sub-criteria and indicators include job creation (total and high-paying jobs created), end-user acceptability (disposal frequency, cleaning requirement, privacy, odor and flies, and security), and property manager acceptability (disposal frequency and cleaning requirement). Compatibility with policy was assumed to be a constraint to system deployment and was not included as a social sub-criterion in the analysis. Any system that was not compatible with policy should automatically be excluded from the analysis before the MCDA is conducted.

Table S23. Social indicators identified in the literature review.

Social & Institutional	Reference(s)
Job creation	19,25,31,32,34
Manageability	32
Compatibility with policy	19,26,32,34
Rapidly accomplishable	32
Socio-cultural/public acceptability	3,5,8,10,15–19,24,25,32,35,36
Consideration for poorest groups of society	19,32
Organizational capacity	22
Visual impact	13,17,21
Government support	13,19
Environmental impact perception	15
EPA/local regulations	2,3
Permitting	2
Population growth	2,35
Equity	19,25
User participation	5,19
Food security	19
Local development	19
Population	27
Convenience	3,13,19,24
Conformity	24
Usability	24
Perception/Complexity of user maintenance	3
Adoptability- the ability of the beneficiary to use the tech	3
Management - oversight	3
Stakeholder engagement	10
Landlord preference	33
User Preference	33

S2.5.1. Job creation sub-criterion indicators

Two indicators are used to characterize job creation: total jobs created and high-paying jobs created (**Table S24**). For specific contexts it may be more influential to create high-paying jobs (skilled jobs), any jobs (unskilled and skilled), or both depending on the unemployment rate and the population below an income level. Since the total number of jobs created is dependent on the number of high-paying jobs created, adjustments to the number of high-paying jobs within the model will automatically adjust the total jobs created. Job requirements for each alternative were determined in Trimmer et al.[43]

Total jobs created (S1). The total jobs created is evaluated as the total skilled and unskilled jobs needed for an alternative. Alternatives A and C require 12 total employees and Alternative B requires 5 to 15 total employees. A uniform distribution was used in simulation of Alternative B's total jobs created.

High-paying jobs created (S2). The high-paying jobs created is evaluated as the total skilled jobs needed for an alternative. In the illustration, Alternatives A and C do not require additional skilled employees and Alternative B requires an additional 5 skilled employees.

Table S24. Job creation indicator scores. Minimum and maximum values are reported for total jobs created for Alternative B.

Indicator	Unit	Alternative A	Alternative B	Alternative C
Total jobs created	unitless	12<br>(constant)	5 - 15<br>(uniform distribution)	12<br>(constant)
High-paying jobs created	unitless	0<br>(constant)	5<br>(constant)	0<br>(constant)

S2.5.2. End-user acceptability sub-criterion indicators

The end-user acceptability sub-criterion is characterized with five indicators: disposal frequency, cleaning requirement, privacy, odor and flies production, and security. Indicator scores were determined by the community survey conducted in Trimmer et al.[43]

Disposal frequency (S3). This indicator represents the number of times per year that the end-user needs to dispose of the sludge in the storage container (**Table S25**). The more times a user must dispose of the waste, the higher the system maintenance is for the end-user. In this example, there are two storage containers: pit latrine and container-based sanitation. The pit latrine (Alternatives A and B) requires emptying every 0.8 (0.3 - 2.4) years, which is converted to emptying 1.25 (0.4 - 3.3) times per year. The container-based system requires emptying every 3.5 (1 - 9) days, which is converted to emptying 104.3 (40.6 - 365) times per year.

Table S25. End-user disposal frequency indicator scores. Midpoint, minimum, and maximum are reported for all triangular distributions.

Indicator	Unit	Alternative A	Alternative B	Alternative C
Disposal frequency	Emptying per year	1.25 [0.4 - 3.3] (triangular distribution)	1.25 [0.4 - 3.3] (triangular distribution)	104.3 [40.6 – 365] (triangular distribution)

Cleaning requirement (S4). High cleaning requirements are often reported as dissatisfactory in a sanitation system. Within a sanitation system, users are often responsible for maintaining and cleaning the user interface. A scale was created that characterized the cleaning requirements based on the reported maintenance for each user interface in the Compendium of Sanitation Systems and Technologies.[45] Urine-diverting dry (UDDT) and flush (UDFT) toilets are the most difficult to keep clean because users may have difficulty separating both streams perfectly, which may result in extra cleaning and maintenance, with water-based diversion systems slightly easier to maintain. Dry toilets and pit latrines do not require additional education and acceptance to be used correctly and are not prone to the clogging or misuse found in UDDT and UDFT systems, so they have a lower burden of cleaning and maintenance on the user. Finally, pour flush toilets and cistern flush toilets have the easiest cleaning requirement, with pour flush toilets being a little more difficult to keep clean and maintain due to the requirement for dry cleansing materials to be collected separately and not flushed down the toilet. As a result, a UDDT is given a score of 1 as it requires the most cleaning and the cistern flush toilet is given a score of 5 as it requires the least amount of cleaning (**Table S26**).

Table S26. Indicator score descriptions to assess end-user cleaning requirement and their application to Alternatives A - C.

Indicator Score	Description	Example	Alternative A	Alternative B	Alternative C
1	Requires frequent cleaning with wiping down the toilet with a mild acid and/or hot water to prevent build-up of mineral deposits and scaling.	Urine Diverting Dry Toilet			X
2	Requires cleaning with a mild acid and/or hot water to prevent build-up of mineral deposits and scaling .	Urine Diverting Flush Toilet			
3	Requires to be cleaned and dried for pathogen removal.	Dry Toilet or Pit			
4	Requires regular cleaning with wiping down the bowl.	Pour Flush Toilet	X	X	
5	Easy to clean and operate with minimal scrubbing of the bowl.	Cistern Flush Toilet			

Privacy (S5). Privacy in sanitation helps vulnerable populations feel more secure when using the sanitation system and empowers users to better maintain and clean their toilets. Some communities value privacy higher than other communities. While privacy can make a system more acceptable for an end-user, it may come with tradeoffs, such as costs. Privacy was quantified using the number of households sharing a toilet (**Table S27**). In this illustration, all three alternatives assume 3 to 5 households are sharing a single pit latrine, flush toilet, or UDDT. Although the indicator scores were constant across all three alternatives in the illustration, decision-makers and technology developers could choose to evaluate sanitation value chains with varied number of households sharing a toilet (e.g., comparing an alternative in which each household gets their own toilet against another alternative with households sharing a single toilet).

Table S27. End-user privacy indicator scores. Minimum and maximum values are reported for all uniform distributions.

Indicator	Unit	Alternative A	Alternative B	Alternative C
Households sharing a toilet	households	3 - 5 (uniform distribution)	3 - 5 (uniform distribution)	3 - 5 (uniform distribution)

Production of odors and flies (S6). The production of odor and flies is influenced by the type of user interface and storage.[45] A scale was created to qualitatively score how a system might produce odors and flies. The compendium describes pit latrines/dry toilets as having noticeable odors even if equipped with a vent pipe and container-based options as having no real problems with odors and vectors (flies) if used and maintained correctly. As a result, pit latrines and dry toilets were assumed to score worse than container-based options with flush systems performing the best. A system with a high production of odors and flies was given a low score (e.g., a non-ventilated pit latrine with a dry toilet = 1) and a system that produced minimal odor and flies was given a high score (e.g., any flush toilet with proper ventilation = 5) (**Table S28**). DMsan users could also modify the scoring based on their applications (e.g., modify it to be a

three-point scale with any non-ventilated toilets scoring 1, ventilated toilets scoring 2, and flush toilets scoring 3 if there is no difference between container-based and pit latrine odors and flies).

Table S28. Indicator score descriptions to assess production of odors and flies and their application to Alternatives A - C.

Indicator Score	Description	Example	Alternative A	Alternative B	Alternative C
1	Storage lacks ventilation and feces is visible (often when lacking the ability to flush).	Non-ventilated pit latrine with a dry toilet			
2	Feces may not be as visible but still has odor due to lack of ventilation.	Non-ventilated container with UDDT			
3	Has some ventilation but lacks flushing and separation of feces and liquids.	Ventilated pit latrine with dry toilet			
4	Has some ventilation and separation of feces that may be less visible, but lacks flushing of feces and liquids.	Ventilated container with UDDT			X
5	Has ventilation and feces is not visible.	Any flush toilet with proper ventilation	X	X	

Security (S7). Security is characterized as the distance a user must travel to use the sanitation system. In this illustration it is assumed the distance travelled does not change among the alternatives, making the indicator not applicable; however, decision-makers and technology developers can choose to vary the average distance between households and toilets across evaluation scenarios.

S2.5.3. Property manager acceptability sub-criterion indicators

Property manager acceptability sub-criterion indicators should be included in the analysis if the user interface is not owned by the user or community member. Disposal frequency and cleaning are the responsibility of the manager instead of the toilet user.

Disposal frequency (S8). Management disposal frequency represents the number of times per year the property manager or owner of the system needs to empty the user interface. The more times a property manager disposes of waste, the higher the system maintenance. This indicator is characterized in the same manner as end-user disposal frequency, except the manager or owner is responsible instead of the toilet user. For this example, the user is responsible for maintaining the toilet, so this indicator was excluded.

Cleaning requirement (S9). Cleaning requirement is characterized in the same manner as end-user cleaning requirement, except the manager or owner is responsible instead of the toilet user. The scores outlined in **Table S26** can be used for this indicator if applicable (i.e., the toilet is not owned by the toilet user). For this example, the user is responsible for cleaning the toilet, so this indicator was excluded.

S3. Determination of indicator weights

For each indicator there is a corresponding indicator weight determined by the contextual driver (importance) of that indicator. The indicator weight for each indicator is calculated using analytical hierarchy process (AHP) pair-wise comparison criteria matrices. The contextual driver score for each indicator is normalized on a scale of 1 to 100 and compared against the other contextual driver scores within its criterion matrix. The contextual driver scores are built within DMsan (location.xlsx) and depend on context-specific information, such as the economic, ecological, and cultural landscape. Below is the description of each indicator contextual driver and the scores assigned for Uganda as well as a description of how the indicator weights are calculated using AHP. Environmental and economic indicators do not have contextual drivers because it is assumed that the environmental indicators are given equal, uniform weights (1/3 weight each) and the economic indicator is given a weight of 1.0 as it is the only indicator within the economic criterion.

S3.1. Technical indicator contextual drivers

S3.1.1. Resiliency and robustness contextual drivers

Extent of training (T1). The extent of training drives how important the indicator user interface robustness is for sanitation system selection. Failures can arise at the user interface when a community does not adequately invest in people to train end-users on how to properly use the system, including what can go in the toilet (e.g., feces, urine, paper products, water), how to clean the toilet, and other maintenance requirements. The World Economic Forum indicator 6.02 Extent of Staff Training is used for this contextual driver.[37] Extent of staff training is the extent of training that companies invest into their employees for each country and is scored on a scale of 1 to 7. The lower this score is, the higher the weight it will have in picking a simpler user interface that requires minimal training. For this example, Uganda has a score of 3.6 out of 7.

Population without at least basic sanitation (T2). The population of a country without at least basic sanitation was selected as a contextual driver to represent the relative importance of resiliency of treatment type for sanitation system selection. Treatment type resiliency will be weighted higher in countries with high populations without at least basic sanitation because system failure in a community with widespread sanitation services is assumed to be less detrimental than in a community with low access to sanitation and no other options. The sanitation coverage data reported by World Health Organization (WHO) and the United Nations Children's Fund (UNICEF) is used to inform this indicator weight calculation.[38] At the time of the report (2020), 80% of the people in Uganda did not have access to at least basic sanitation facilities.

S3.1.2. Feasibility contextual drivers

Technology absorption (T3). Technology absorption was selected to describe the importance of the indicator accessibility to parts for sanitation system selection. A country's absorption of the latest technology could influence a community's ability to support a more advanced sanitation system that requires significant custom parts. Parts can be shipped to the community but could limit the ability of a community to replace parts as the system is operated and maintained. Countries with higher levels of advanced technology were assumed to have better access to custom parts that may be required for novel and advanced systems: more access to custom parts resulted in lower importance of accessibility to parts because all parts are viewed as accessible. The World Economic Forum indicator 9.02 Firm-level Technology Absorption rates

the extent of businesses adopting the latest technologies on a scale of 1 to 7 with low scores representing low technology absorption rates.[37] For this example, Uganda has a score of 4.0 out of 7 in technology absorption.

Quality of roads (T4). Quality of roads drives how important the indicator transportation feasibility is for sanitation system selection. The quality of roads can influence the type of conveyance system that can be implemented for a sanitation system. The World Economic Forum indicator 2.02 Quality of Roads rates the road qualities within a country on a scale of 1 to 7 with low scores representing extremely poor road conditions.[37] Uganda has a score of 3.4 out of 7 in quality of roads.

Construction skills available (T5). Construction skills available drives how important the indicator construction skills required is for sanitation system selection. The score for this contextual driver is based on the fraction of the workforce employed in the construction field.[39] Uganda's construction workforce makes up 2.1% of the entire workforce. Across all countries in the database, the maximum fraction of the workforce employed in the construction field is 40.5%. To determine an overall score for this contextual driver, the country-specific fraction was divided by the maximum fraction (i.e., 2.1/40.5 * 100), thus the score for Uganda is 5.2 out of 100.

Professional skills available (T6). Professional skills available drives how important the indicator operation and maintenance skills required is for sanitation system selection. The World Economic Forum indicator 12.06 Availability of Scientists and Engineers rates the extent at which scientists and engineers are available on a scale of 1 to 7 with low scores representing unavailable professionals.[37] Uganda has a score of 4.1 out of 7.

S3.1.3. Flexibility contextual drivers

Population growth rate (T7). The population growth rate drives how important the indicator population flexibility is for sanitation system selection. The population growth rate for Uganda is 3.6%.[40] Across all countries in the database, the maximum growth rate is 4.5% and the minimum growth rate is -1.8%. To determine an overall score for this contextual driver, the country-specific growth rate was divided by the difference between the maximum and minimum growth rates (i.e., 3.6/[4.5 - [-1.8]] * 100), thus the score for Uganda is 57.1 out of 100.

Electricity coverage (T8). The electricity coverage drives how important the indicator power outage flexibility is for sanitation system selection. The contextual driver is based on the power outages in firms in a typical month reported by The World Bank.[40] Uganda has 6.3 blackouts per month. Across all countries in the database, the maximum number of blackouts per month is 75.2. To determine an overall score for this contextual driver, the country-specific fraction was divided by the maximum fraction (i.e., 6.3/75.2 * 100), thus the score for Uganda is 8.4 out of 100.

Baseline water stress (T9/RR1). The baseline water stress drives how important the indicator drought flexibility is for sanitation system selection. The baseline water stress, reported by the World Resources Institute, measures the ratio of total water withdrawals available to renewable water supplied, where high values indicate more competition for water among users (i.e., more water stress).[41] The baseline water stress in Uganda is 0.26. Across all countries in the database, the maximum baseline water stress is 4.82. To determine an overall score for this contextual driver, the country-specific baseline water stress was divided by the maximum baseline water stress (i.e., 0.26/4.82 * 100), thus the score for Uganda is 5.4 out of 100.

S3.2. Resource recovery indicator contextual drivers

Baseline water stress (T9/RR1). As described in the previous section, the baseline water stress drives how important the indicator water recovery is for sanitation system selection. This contextual driver is used to develop indicator weights for both drought flexibility (T9) and water recovery (RR1).

Nitrogen fertilizer fulfillment (RR2). Nitrogen fertilizer fulfillment drives how important the indicator nitrogen recovery is for sanitation system selection. This contextual driver is calculated as the ratio between the nitrogen fertilizers used and the nitrogen fertilizers needed based on crop production in the country. The World Bank reports the fertilizer use by country as Fertilizers by Nutrient and the crop production within a country as Crops and Livestock Products.[40] Nitrogen fertilizer need is calculated using the crop production and the recommended fertilizer application by crop.[42] The calculated Uganda nitrogen fertilizer fulfillment 1.4% out of 100%.

Phosphorus fertilizer fulfillment (RR3). Like nitrogen fertilizer fulfillment, phosphorous fertilizer fulfillment drives how important the indicator phosphorous recovery is for sanitation system selection. The calculated Uganda phosphorous fertilizer fulfillment is 1.1% out of 100%

Potassium fertilizer fulfillment (RR4). Like nitrogen and phosphorous fertilizer fulfillment, potassium fertilizer fulfillment drives how important the indicator potassium recovery is for sanitation system selection. The calculated Uganda potassium fertilizer fulfillment is 0.5% out of 100%.

Renewable energy consumption (RR5). Renewable energy consumption drives how important the indicator energy recovery is for sanitation system selection. This contextual driver is based on the renewable energy consumption (% of total final energy consumption) reported by The World Bank.[40] Uganda uses 89% renewable energy for their total energy consumption.

Infrastructure quality (RR6). Infrastructure quality drives how important the indicator supply chain feasibility is for sanitation system selection. The World Economic Forum indicator 2.01 Quality of Overall Infrastructure rates the general state of infrastructure (e.g., transportation, communications, and energy) on a scale of 1 to 7 with low scores representing extremely underdeveloped infrastructure.[37] Uganda has a score of 3.3 out of 7 for overall infrastructure quality.

S3.3. Environmental and economic indicator contextual drivers

There are no contextual drivers for the environmental and economic indicators. It is assumed that the indicator weights are equal (1/3) for the three environmental indicators (Env1, Env2, and Env3), and no indicator weight is necessary for the single economic indicator (Econ1).

S3.4. Social indicator contextual drivers

S3.4.1. Job creation contextual drivers

Unemployment rate (S1). Unemployment rate drives how important the indicator total jobs created is for sanitation system selection. A social benefit of sanitation infrastructure is the number of jobs that it can create, especially for communities who have high unemployment rates.

The World Bank reports the unemployment total (% of total labor force),[40] and in Uganda, the unemployment rate is 2.4%.

International poverty line (S2). The international poverty line drives how important the indicator high-paying jobs created is for sanitation system selection. Although job creation in any capacity can help communities facing unemployment challenges, high-paying jobs are especially important in communities with a high percentage of their employed population earning below the international poverty line ($\$1.90{\bullet}day^{-1}$). The International Labour Organization reports the percentage of employed individuals earning below $\$1.90{\bullet}day^{-1}$,[39] and in Uganda, the percentage of employed individuals living below $\$1.90{\bullet}day^{-1}$ is 35.1%.

S3.4.2. End-user acceptability contextual drivers

It can be difficult to determine community-specific indicator weights without surveying the individuals using the sanitation system. A survey of members of the Bwaise community evaluated which factors negatively affect their perception of sanitation systems.[43] Summarized results from the household survey in Bwaise and example survey questions can be found in the Trimmer et al. Supplementary Information. The community survey revealed that factors that cause dissatisfaction of the system include unclean facilities (66%), long waiting times (25%), fear for personal safety (24%), and limited privacy (16%). These values were used to calculate the related social indicator weights, and future research could develop community survey questions that explicitly allow toilet users to rank and input their preferences related to social indicators.

Disposal preference (S3). In the survey, the community did not report any preference or dissatisfaction with the number of times to dispose of the waste in the storage container. As a result, no contextual driver score was assigned for this indicator.

Cleaning preference (S4). Two social indicator contextual drivers relate to unclean facilities: cleaning preference and odor and flies preference. Because it is unclear which is driving unclean facilities, it was assumed that 2/3 of the survey members report unclean facilities due to cleaning preference and 1/3 of the survey members report unclean facilities due to odor and flies present. The contextual driver score of 44 out of 100 (calculated as 2/3 * 66) was assigned to account for some of the respondents also being concerned with odors and flies.

Privacy preference (S5). In the survey, a large portion of the community members reported that long waiting times, safety, and unclean facilities were a cause for dissatisfaction with their sanitation facility. More private toilets (shared by one or two households) could reduce the amount of waiting times, safety concerns, and unclean facilities. Furthermore, communities that feel ownership over their user interface and storage are more likely to maintain the system better. The community percentage of long waiting times and limited privacy were summed together (i.e., 25 + 16) to obtain a score of 41 out of 100 for this contextual driver.

Odor and flies preference (S6). As discussed in the cleaning preference contextual driver, odor and flies could be a contributing factor to dissatisfaction in facility cleanliness. It was assumed 1/3 of survey respondents reporting unclean facilities are dissatisfied due to odor and flies. A score of 22 out of 100 (calculated as 1/3 * 66) was given for this contextual driver.

Security (S7). The contextual driver for end-user acceptability is security. This indicator is a critical factor in sustainable and safe sanitation systems as unsecure facilities can lead to psychological stress, threats of violence, and health issues. Since 24% of the population surveyed reported personal safety as a concern, a score of 24 out of 100 was used for this contextual driver.

S3.4.3. Property manager acceptability contextual drivers

Like the end-user acceptability sub-criteria, it is difficult to weigh the landlord's perception of sanitation systems without having some information from the specific management. It is encouraged to gather information to adequately assess the preference disposal and/or cleaning. Bwaise end-users are responsible for requesting disposal and cleaning the sanitation units, thus, management values were not applicable for this example.

Disposal preference (S8). For this example, this contextual driver was not applicable.

Cleaning preference (S9). For this example, this contextual driver was not applicable

S3.5. The analytical hierarchy process (AHP) for calculating indicator weights

The following steps are used to calculate the indicator weights for technical, resource recovery, and social indicators. It is assumed that environmental indicators use uniform weights (i.e., 1/3 weight for each indicator) and economic uses an indicator weight of 1.0 for its single indicator (annual cost per capita).

To calculate the technical, resource recovery, and social indicator weights, tables are produced for each of the three criteria containing indicator weight scores. Then within each table, the contextual driver scores are normalized on a scale out of 100, and a pair-wise comparison matrix is formed by dividing each row value by the column value. The pair-wise comparison matrix is normalized to calculate the approximate eigenvector values (v_i) based on the position values (a_{ij}) and number of array elements (n) representing the number of indicators (**Equation S1**). The criteria weight vectors (w_i) are calculated by taking the average of each vector row in the normalized pair-wise comparison matrix (**Equation S2**).

$$v_i = \prod_{j=1}^{n} a_{ij}^{\frac{1}{n}}$$ **Equation S1**

$$w_i = \frac{v_i}{\sum_{i=1}^{n} v_i}$$ **Equation S2**

Finally, to check for consistency, the weighted sum value is calculated by multiplying each row of the original pair-wise comparison matrix by the column indicator weight and dividing the product by the indicator weight for the specific row. Then, λ_{max} is calculated by taking the average of the weighted sum value (**Equation S3**). The consistency index (CI) is estimated by subtracting the number of indicators from λ_{max} and dividing it by one less than the number of indicators (**Equation S4**). The random consistency index (RI) is assigned based on the size of the matrices. The consistency ratio (CR) is the quotient of CI over the (RI) (**Equation S5**). Once the CR is calculated, the matrix is consistent if CR is less than or equal to 0.1.

$$\lambda_{max} = \sum_{i=1}^{n} C_{ij} w_i$$ **Equation S3**

$$CI = \frac{\lambda_{max} - n}{n - 1}$$ **Equation S4**

$$CR = \frac{CI}{RI}$$ **Equation S5**

The resulting indicator weights (**Table S29**) are used with the overall criteria weights and indicator scores to calculate the performance of each sanitation alternative. Each country included in DMsan will have its own set of indicator weights depending on the contextual driver scores for each context.

Table S29. Uganda-specific indicator weights.

Criteria	Sub-criteria	Indicator	Indicator Weight
Technical	Resiliency & robustness	User interface robustness	0.11
		Resiliency of treatment type	0.19
	Feasibility	Accessibility to parts	0.10
		Transportation feasibility	0.12
		Construction skills required	0.22
		Operation and maintenance skills required	0.10
	Flexibility	Population flexibility	0.13
		Power outage flexibility	0.02
		Drought flexibility	0.01
Resource recovery	Resource recovery feasibility	Water recovery	0.01
		Nitrogen recovery	0.23
		Phosphorous recovery	0.23
		Potassium recovery	0.23
		Energy recovery	0.20
		Supply chain feasibility	0.10
Environmental	Life cycle environmental impacts	Damage to ecosystems	0.33
		Damage to human health	0.33
		Damage to resources	0.33
Economic	Net costs	Annual cost per capita	1.00
Social	Job creation	Total jobs created	0.05
		High-paying jobs created	0.20
	End-user acceptability	Disposal frequency	0.24
		Cleaning requirement	0.26
		Privacy	0.12
		Odor and flies	0.13
		Security	0.00
	Property manager acceptability	Disposal frequency	0.00
		Cleaning requirement	0.00

S4. Multi-criteria decision analysis methods to evaluate sanitation alternatives

There are four steps in MCDA: (1) select criteria and indicators, (2) assign criterion and indicator weights, (3) determine indicator scores, and (4) calculate performance scores of each alternative. The objective is to select the most appropriate sanitation system based on the local context. The criteria in DMsan include technical, resource recovery, environmental, economic, and social with sub-criteria and indicators based on the trends identified in the literature review (**Section S1**). Indicator weights were determined within criteria matrices by using the analytical hierarchy process (AHP). Additionally, for the Bwaise illustration, 1,000 criteria weight scenarios were simulated to assess sanitation system performance under the entire spectrum of stakeholder preferences (**Section S4.1**); however, users can simulate as many or little scenarios as desired. Finally, alternatives can be ranked using technique for order by preference of similarity (TOPSIS) calculations that incorporate indicator scores (**Section S2**) indicator weights (**Section S3**), and criteria weights.

S4.1. Criteria weight scenarios

Due to the lack of and subjective nature of expert-informed weighting, criteria weight scenarios can be simulated within DMsan to evaluate the entire spectrum of weight options for each criterion (criterion weight ranges from 0 to 1). When a criterion has a weight of 0, all indicators related are not included in the decision. Likewise, when a criterion has a weight of 1, only its supporting indicators are included in the decision. If project has stakeholder- or expert-informed criterion weights, technology developers and decision-makers can modify the code within DMsan to use this set of criteria weights instead of the simulated criteria weight scenarios. DMsan users can simulate as many criterion weights as desired. For the Bwaise illustration, 1,000 criterion weight scenarios were simulated to capture the full spectrum of the decision space (**Figure S1**).

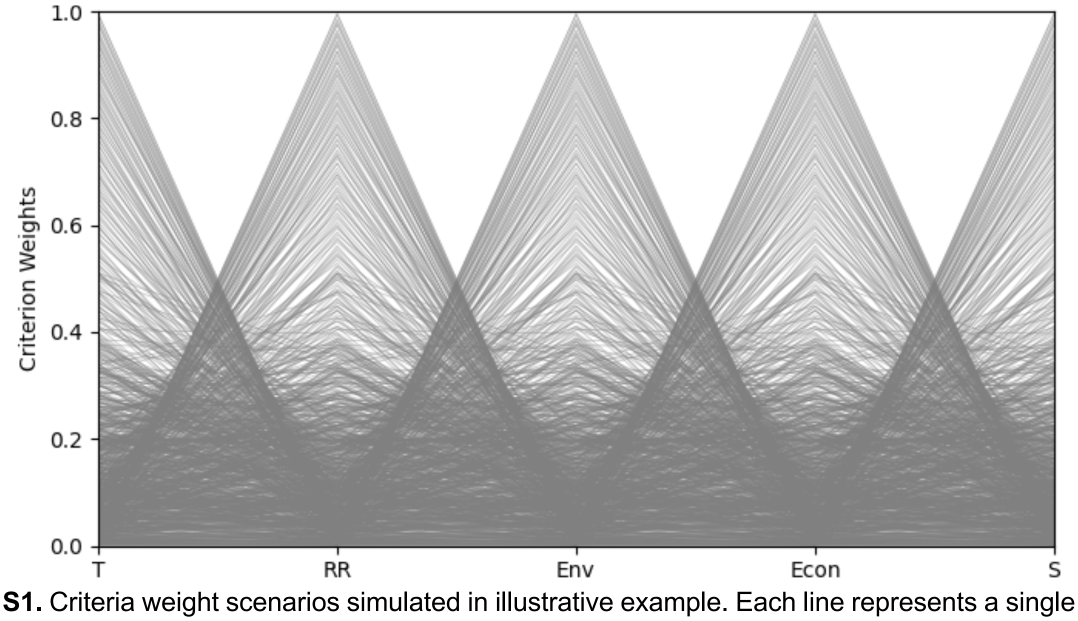

Figure S1. Criteria weight scenarios simulated in illustrative example. Each line represents a single criteria weight scenario with y-values representing the individual criterion weight for each of the five criteria. Criteria weights range from 0 (i.e., the criterion is not included in the decision) to 1 (i.e., only the specific criterion is important for decision-making).

S4.2. The technique for order by preference of similarity (TOPSIS) method for calculating performance score and rank of sanitation alternatives

The following steps are used to calculate performance scores for each sanitation system alternative. First, a decision matrix (A_{ij}) is created where i is a particular alternative, m is the total number of alternatives (m = 3), j is a particular indicator, and n is the total number of indicators (n = 28). Each value in the decision matrix is an indicator score (X_{ij}). The indicators are categorized as "beneficial", indicating a higher indicator score is best (e.g., total jobs created), or "non-beneficial", indicating a lower indicator score is best (e.g., annual cost per capita). Each indicator score is normalized using vector normalization (**Equation S6**). Each value in the normalized decision matrix is a normalized indicator score. Next, the weighted normalized decision matrix is created by multiplying each normalized indicator score by the indicator weight ($\underline{IW_j}$) and the criteria weight (CW_k where k is the criterion) (e.g., the energy recovery normalized indicator score is multiplied by the energy recovery indicator weight and the resource recovery criterion weight) (**Equation S7**).

$$\bar{X}_{ij} = \frac{X_{ij}}{\sqrt{\sum_{i=1}^{m} X_{ij}^2}} \quad \textbf{Equation S6}$$

$$\bar{\bar{X}}_{ij} = \bar{X}_{ij} \times IW_j \times CW_k \quad \textbf{Equation S7}$$

Next, the ideal best (V_j^+) and ideal worst (V_j^-) are identified for each indicator. For beneficial indicators, the ideal best is the maximum weighted normalized indicator score among the alternatives, and the ideal worst is the minimum weighted normalized indicator score among the alternatives. Likewise, for non-beneficial indicators, the ideal best is the minimum weighted normalized indicator score, and the ideal worst is the maximum weighted normalized indicator score. Once the ideal best and ideal worst have been identified for each indicator, the Euclidean distance is calculated from the ideal best (S_i^+) and ideal worst (S_i^-) for each alternative (**Equation S8, Equation S9**).

$$S_i^+ = [\sum_{j=1}^{n} \left(\bar{\bar{X}}_{ij} - V_j^+\right)^2]^{0.5} \quad \textbf{Equation S8}$$

$$S_i^- = [\sum_{j=1}^{n} \left(\bar{\bar{X}}_{ij} - V_j^-\right)^2]^{0.5} \quad \textbf{Equation S9}$$

Finally, the performance score (P_i) of each alternative is calculated using the Euclidean distances from the ideal best and ideal worst (**Equation S10**). The best performing alternative is the alternative with the highest performance score.

$$P_i = \frac{S_i^-}{S_i^+ + S_i^-} \quad \textbf{Equation S10}$$

S5. System simulation with techno-economic analysis and life cycle assessment

Simulation, techno-economic analysis (TEA), and life cycle assessment (LCA) for each alternative were performed in Python using QSDsan. Design and TEA of the alternatives follow algorithms and assumptions used in Trimmer et al.[43] Although not used in this analysis, technology developers interested in converting costs from one country to another can incorporate the price level ratios by country found in the contextual parameter database for the package, or they can set specific contextual parameter values in QSDsan if local data are available. For LCA, the life cycle impact assessment method of ReCiPe (the H ["hierarchist"] perspective)[48] was used instead of TRACI in the original paper; however, DMsan users could change the method depending on the desired environmental impact indicator (e.g., using TRACI to include eutrophication potential). Any or all indicators for a given LCIA method can be used as individual indicators in DMsan. It should be noted that although total life cycle environmental impacts were assessed for each system, DMsan users could break down LCA results by sanitation unit process or life cycle stage within QSDsan, if desired, to identify problem areas for different types of damage across the sanitation service chain. Life cycle inventory data were obtained from the ecoinvent database (v3.7.1, at the point of substitution)[49] through BW2QSD,[50] EcoInventDownloader,[51] and BrightWay2.[52] For each impact item (e.g., cement), a keyword string (e.g., "market cement, unspecified") with constraints (e.g., location to be either "GLO" for global, or "RoW" for rest of the world) was used in in BW2QSD to select impact items that satisfy the keyword and constrains from the ecoinvent database, then minimum, mean, and maximum of the impact characterization factors (CFs) of all selected items were used as the lower bound, midpoint (baseline), and upper bound of a triangular distribution for this impact item in uncertainty analysis. The completion list of the keywords and constraints for the impact items can be found online *(_lca_data.py* in the *bwaise* module of EXPOsan).[53]

Complete codes for all Python libraries used in this study,[50–54] the three alternatives,[53] and uncertainty analysis,[53] can be found online. A total of 167, 165, and 152 uncertainty parameters were included for Alternatives A, B, and C, respectively (**Tables S30-S35**). Except for all characterization factors (CFs) used in LCA, all baseline and uncertainty ranges of parameters were from Trimmer et al.[43,53]

Table S30. Parameter values, ranges, and distributions shared by all three alternatives (total=108).

Parameter	Baseline	Distribution[a]	Lower	Midpoint	Upper
Caloric intake [$kcal \cdot cap^{-1} \cdot d^{-1}$]	2130	U	1917	-	2343
Vegetable protein intake [$g \cdot cap^{-1} \cdot d^{-1}$]	40.29	U	36.26	-	44.32
Animal protein intake [$g \cdot cap^{-1} \cdot d^{-1}$]	12.39	U	11.15	-	13.63
N content of protein [%]	13%	U	13%	-	19%
P content of vegetable protein [%]	2.2%	T	0.4%	2.2%	4.8%
P content of animal protein [%]	1.1%	T	0.2%	1.1%	3.2%
K content of caloric intake [$g\ K \cdot 1000\ kcal^{-1}$]	1.2	U	1.1	-	1.5
N excretion [% of intake]	100%	U	99%	-	100%
P excretion [% of intake]	100%	U	99%	-	100%
K excretion [% of intake]	88%	U	65%	-	98%
Energy excretion [% of intake]	6%	U	2%	-	10%
N in urine [% of total]	88%	T	74%	88%	93%
P in urine [% of total]	61%	T	33%	61%	75%
K in urine [% of total]	74%	T	53%	74%	93%
Energy in feces [% of total]	81%	T	69%	81%	90%
Urine N in reduced inorganic form [%]	85%	U	75%	-	90%
Feces N in reduced inorganic form [%]	20%	U	16%	-	24%
Urine excretion [$g \cdot cap^{-1} \cdot d^{-1}$]	1400	T	800	1400	2500
Feces excretion [$g \cdot cap^{-1} \cdot d^{-1}$]	250	T	75	250	520
Urine moisture content [%]	95%	T	93%	95%	97%
Feces moisture content [%]	85%	T	76%	85%	88%
Mg in urine [$g\ Mg \cdot cap^{-1} \cdot d^{-1}$]	0.2	U	0.12	-	0.21

Mg in feces [g Mg·cap^{-1}·d^{-1}]	0.25	U	0.15	-	0.34
Ca in urine [g Ca·cap^{-1}·d^{-1}]	0.28	U	0.057	-	0.5
Ca in feces [g Ca·cap^{-1}·d^{-1}]	1.9	U	0.1	-	3.6
Household size [cap·household^{-1}]	4	N	4	-	1.8
Exchange rate [UGX·USD^{-1}]	3700	T	3600	3700	3900
Household use density [household·toilet^{-1}]	4	U	3	-	5
Max methane emission [g CH_4·g COD^{-1}]	0.25	T	0.175	0.25	0.325
Time to full degradation [y]	2	U	1	-	3
Reduction at full degradation [log unit]	3	U	2	-	4
Plastic sheet mass [kg·m^{-2}]	0.63	U	0.31	-	1.24
Brick volume [kg·m^{-3}]	1750	U	1500	-	2000
Steel sheet mass [kg·m^{-2}]	2.64	U	2.26	-	3.58
Gravel bulk density [kg·m^{-3}]	1600	U	1520	-	1680
Sand bulk density [kg·m^{-3}]	1442	U	1281	-	1602
Steel density [kg·m^{-3}]	7900	U	7750	-	8050
Transportation loss (N, P, K, Mg, Ca, C) [% of input]	2%	U	2%	-	5%
Transportation distance [km]	5	U	2	-	10
Drying bed retention time [d]	180	T	180	180	270
Drying bed maximum COD degradation [% of total]	70%	T	60%	70%	80%
Drying bed MCF decay [% of degraded COD]	20%	T	0%	20%	30%
Drying bed maximum N degradation [% of N removal]	80%	T	70%	80%	90%
Drying bed N_2O emission factor [% of degraded N]	0.5%	T	0%	0.5%	1.1%
Drying bed column height [m]	2.75	U	2.5	-	3
Drying bed steel column mass [kg·m^{-1}]	30	U	23	-	37
Drying bed concrete thickness [m]	0.3	U	0.15	-	0.45
Drying bed cover slope [degree]	20	U	10	-	30
Drying bed cover mass [kg·m^{-2}]	2.64	U	2.26	-	3.58
Drying bed final solids content [%]	35%	U	30%	-	40%
Drying bed wall height [m]	0.6	U	0.45	-	0.75
Drying bed storage bed wall height [m]	1.5	U	1.2	-	1.8
Ammonia transfer loss [% of input ammonia]	5%	U	0%	-	10%
Other transfer losses (non-ammonia N, P, K, Mg, Ca, C) [% of input]	2%	U	0%	-	5%
Plant lifetime [y][b]	8 or 10	T	8 or 9	8 or 10	11
Brick ecosystem quality CF [point·kg^{-1}]	9.88E-03	T	-2.16E-04	9.88E-03	2.60E-02
Brick human health CF [point·kg^{-1}]	1.01E-02	T	-9.77E-04	1.01E-02	2.69E-02
Brick resources CF [point·kg^{-1}]	2.49E-02	T	-6.50E-04	2.49E-02	9.11E-02
Cement ecosystem quality CF [point·kg^{-1}]	1.79E-02	T	1.74E-02	1.79E-02	1.90E-02
Cement human health CF [point·kg^{-1}]	2.69E-02	T	2.69E-02	2.69E-02	2.69E-02
Cement resources CF [point·kg^{-1}]	1.32E-02	T	1.32E-02	1.32E-02	1.34E-02
Concrete ecosystem quality CF [point·m^{-3}]	9.00E+00	T	8.18E+00	9.00E+00	1.02E+01
Concrete human health CF [point·m^{-3}]	1.14E+01	T	1.04E+01	1.14E+01	1.29E+01
Concrete resources CF [point·m^{-3}]	7.98E+00	T	7.23E+00	7.98E+00	9.06E+00
Excavation ecosystem quality CF [point·m^{-3}]	1.08E-02	T	1.06E-02	1.08E-02	1.11E-02
Excavation human health CF [point·m^{-3}]	2.56E-02	T	2.53E-02	2.56E-02	2.60E-02
Excavation resources CF [point·m^{-3}]	2.67E-02	T	2.53E-02	2.67E-02	2.82E-02
Gravel ecosystem quality CF [point·kg^{-1}]	2.85E-04	T	-2.35E-04	2.85E-04	6.97E-04
Gravel human health CF [point·kg^{-1}]	7.23E-05	T	-1.02E-03	7.23E-05	7.13E-04
Gravel resources CF [point·kg^{-1}]	2.31E-04	T	-6.84E-04	2.31E-04	7.90E-04
Plastic liner ecosystem quality CF [point·kg^{-1}]	6.06E-02	T	5.46E-02	6.06E-02	6.67E-02
Plastic liner human health CF [point·kg^{-1}]	8.52E-02	T	7.67E-02	8.52E-02	9.38E-02
Plastic liner resources CF [point·kg^{-1}]	2.23E-01	T	2.01E-01	2.23E-01	2.46E-01
Sand ecosystem quality CF [point·kg^{-1}]	-4.50E-03	T	-2.80E-03	-4.50E-03	-5.52E-03
Sand human health CF [point·kg^{-1}]	-5.93E-04	T	6.13E-05	-5.93E-04	-1.03E-03
Sand resources CF [point·kg^{-1}]	2.18E-03	T	2.30E-03	2.18E-03	1.86E-03
Stainless steel ecosystem quality CF [point·kg^{-1}]	1.72E-01	T	1.67E-01	1.72E-01	1.76E-01
Stainless steel human health CF [point·kg^{-1}]	2.77E-01	T	2.72E-01	2.77E-01	2.82E-01
Stainless steel resources CF [point·kg^{-1}]	7.82E-01	T	7.64E-01	7.82E-01	8.00E-01
Stainless steel sheet ecosystem quality CF [point·kg^{-1}]	2.00E-01	T	1.95E-01	2.00E-01	2.04E-01
Stainless steel sheet human health CF [point·kg^{-1}]	3.08E-01	T	3.03E-01	3.08E-01	3.14E-01
Stainless steel sheet resources CF [point·kg^{-1}]	8.37E-01	T	8.19E-01	8.37E-01	8.55E-01
Steel ecosystem quality CF [point·kg^{-1}]	6.35E-02	T	5.65E-02	6.35E-02	7.13E-02
Steel human health CF [point·kg^{-1}]	7.75E-02	T	6.84E-02	7.75E-02	8.46E-02
Steel resources CF [point·kg^{-1}]	3.96E-01	T	2.85E-01	3.96E-01	5.82E-01
Wood ecosystem quality CF [point·m^{-3}]	1.17E+02	T	1.12E+02	1.17E+02	1.21E+02
Wood human health CF [point·m^{-3}]	5.56E+00	T	4.44E+00	5.56E+00	7.16E+00

Wood resources CF [point·m^{-3}]	4.35E+00	T	3.77E+00	4.35E+00	5.06E+00
Truck ecosystem quality CF [point·tonne^{-1}·km^{-1}]	3.50E-03	T	3.41E-03	3.50E-03	3.63E-03
Truck human health CF [point·tonne^{-1}·km^{-1}]	5.65E-03	T	5.29E-03	5.65E-03	6.37E-03
Truck resources CF [point·tonne^{-1}·km^{-1}]	7.19E-03	T	6.94E-03	7.19E-03	7.48E-03
N fertilizer ecosystem quality CF [point·kg N^{-1}]	-2.43E+03	T	-9.73E+03	-2.43E+03	-2.46E-01
N fertilizer human health CF [point·kg N^{-1}]	-2.35E-01	T	-2.64E-01	-2.35E-01	-1.83E-01
N fertilizer resources CF [point·kg N^{-1}]	-2.37E-01	T	-3.09E-01	-2.37E-01	-1.03E-01
P fertilizer ecosystem quality CF [point·kg P^{-1}]	-3.97E+03	T	-1.59E+04	-3.97E+03	-5.64E-01
P fertilizer human health CF [point·kg P^{-1}]	-5.95E-01	T	-7.98E-01	-5.95E-01	-4.15E-01
P fertilizer resources CF [point·kg P^{-1}]	-4.83E-01	T	-5.78E-01	-4.83E-01	-2.94E-01
K fertilizer ecosystem quality CF [point·kg K^{-1}]	-6.50E+02	T	-2.60E+03	-6.50E+02	-1.77E-01
K fertilizer human health CF [point·kg K^{-1}]	-2.10E-01	T	-3.09E-01	-2.10E-01	-1.56E-01
K fertilizer resources CF [point·kg K^{-1}]	-1.69E-01	T	-2.26E-01	-1.69E-01	-9.55E-02
Electricity ecosystem quality CF [point·kWh^{-1}]	4.57E-03	T	1.05E-04	4.57E-03	2.12E-02
Electricity human health CF [point·kWh^{-1}]	6.47E-03	T	2.03E-04	6.47E-03	3.34E-02
Electricity resources CF [point·kWh^{-1}]	6.48E-03	T	2.40E-04	6.48E-03	3.64E-02
Sludge fertilizer discount factor [price in sludge·price in commercial fertilizer^{-1}]	0.25	U	0.1	-	0.4
N fertilizer price [USD·kg N^{-1}]	1.51	U	1.16	-	2.30
P fertilizer price [USD·kg P^{-1}]	3.98	U	2.62	-	6.69
K fertilizer price [USD·kg K^{-1}]	1.33	U	1.21	-	1.47
Discount rate [%]	0.05	U	0.03	-	0.06
Electricity price [$·kWh^{-1}]	0.17	T	0.08	0.17	0.21

[a] U, T, and N represent uniform, triangular, and normal distribution, respectively. For normal distribution, the lower value is μ and the upper value is sigma.

[a] When two values are presented, the first value is for the existing plant and the second value is for the alternative plant.

Table S31. Parameter values, ranges, and distributions shared by Alternatives A and B (total=28).

Parameter	Baseline	Distribution[b]	Lower	Midpoint	Upper
Pit latrine toilet paper usage [kg·cap^{-1}·h^{-1}]	2.82E-04	U	2.49E-04	-	3.42E-04
Pit latrine flushing water usage [kg·cap^{-1}·h^{-1}]	0.42	T	0.17	0.42	1.04
Pit latrine cleansing water usage [kg·cap^{-1}·h^{-1}]	0.042	U	0.015	-	0.13
Pit latrine desiccant volume [m^3·cap^{-1}·h^{-1}]	8.33E-06	T	8.33E-06	8.33E-06	2.08E-05
Pit latrine maximum COD removal [% of input]	70%	T	60%	70%	80%
Pit latrine maximum N degradation [% of input]	80%	T	70%	80%	90%
Pit latrine MCF aquatic [% of degraded COD]	11%	T	0.4%	11%	27%
Pit latrine N_2O aquatic emission factor [% of degraded N]	0.5%	T	0.1%	0.5%	7.5%
Pit latrine desiccant density [kg·m^{-3}][a]	760	T	663	760	977
Pit latrine desiccant Mg content [%][a]	0.042	U	0.015	-	0.13
Pit latrine desiccant Ca content [%][a]	8.33E-06	T	8.33E-06	8.33E-06	2.08E-05
Pit latrine emptying period [y]	0.8	T	0.3	0.8	2.4
Pit latrine sludge accumulation rate [L·cap^{-1}·y^{-1}]	270	T	100	270	900
Pit latrine N leaching [% of input]	13%	U	1%	-	50%
Pit latrine P leaching [% of input]	18%	U	0%	-	37%
Pit latrine K leaching [% of input]	21%	U	11%	-	31%
Pit latrine N volatilization [% of input]	0.5%	U	0%	-	1%
Pit latrine MCF decay [% of degraded COD]	50%	T	40%	50%	60%
Pit latrine N_2O emission factor [% of degraded N]	0%	T	0%	0%	0.1%
Pit latrine capital cost [USD·toilet^{-1}]	449	U	386	-	511
Pit latrine annual operating cost [% of capital]	5%	U	2%	-	8%
Tanker truck additional emptying fee [% of base cost]	15%	U	0%	-	30%
Sedimentation tank final solids content [%]	14%	U	10%	-	20%
Sedimentation tank/sludge separator solids retention [% of input]	50%	U	35%	-	60%
Sedimentation tank/sludge separator COD retention [% of input]	50%	U	35%	-	60%
Sedimentation tank/sludge separator N retention [% of input]	6%	T	2%	6%	16%
Sedimentation tank/sludge separator P retention [% of input]	20%	T	9%	20%	40%
Sedimentation tank/sludge separator K retention [% of input]	13%	T	2%	13%	28%
Sedimentation tank/sludge separator Mg retention [% of input]	28%	U	19%	-	37%
Sedimentation tank/sludge separator Ca retention [% of input]	44%	U	22%	-	53%

[a] The pit latrine unit in QSDsan allows the user to choose whether or not to add desiccant. No desiccant was added in the simulation for this study according to Trimmer et al.

[b] U, T, and N represent uniform, triangular, and normal distribution, respectively. For normal distribution, the lower value is μ and the upper value is sigma.

[b] When two values are presented, the first value is for the existing plant and the second value is for the alternative plant.

Table S32. Parameter values, ranges, and distributions shared by Alternatives A and C (total=16).

Parameter	Baseline	Distribution[a]	Lower	Midpoint	Upper
Sewered population served by existing plant	40000	U	30000	-	50000
Population producing latrine sludge treated by existing plant	416667	T	416667	416667	458333
Anaerobic lagoon COD removal [% of input]	70%	T	60%	70%	80%
Anaerobic lagoon maximum COD degradation [% of retained]	70%	T	60%	70%	80%
Anaerobic lagoon MCF decay [% of degraded COD]	80%	T	80%	80%	100%
Anaerobic lagoon maximum denitrification [% of N removal]	80%	T	70%	80%	90%
Anaerobic lagoon N_2O emission factor [% of degraded N emitted as N_2O]	0%	U	0%	-	0.1%
Anaerobic lagoon plastic liner mass [kg·m^{-2}]	0.63	U	0.31	-	1.24
Anaerobic lagoon sewer flow [m^3·d^{-1}]	2750	U	2500	-	3000
Facultative lagoon COD removal [% of input]	70%	T	70%	70%	90%
Facultative lagoon maximum COD degradation [% of retained]	70%	T	60%	70%	80%
Facultative lagoon MCF decay [% of degraded COD]	20%	T	0%	20%	30%
Facultative lagoon maximum N degradation [% of input]	80%	T	70%	80%	90%
Facultative lagoon N_2O emission factor [% of degraded N]	1.6%	T	0%	1.6%	4.5%
Facultative lagoon P removal [% of input]	60%	U	50%	-	70%
Facultative lagoon plastic liner mass [kg·m^{-2}]	0.63	U	0.31	-	1.24
Facultative lagoon sewer flow [m^3·d^{-1}]	2750	U	2500	-	3000
Salary for existing plant staff [million UGX·cap^{-1}·month^{-1}]	3	U	1	-	5

[a] U, T, and N represent uniform, triangular, and normal distribution, respectively. For normal distribution, the lower value is μ and the upper value is sigma.

[a] When two values are presented, the first value is for the existing plant and the second value is for the alternative plant.

Table S33. Parameter values, ranges, and distributions unique to Alternative A (total=9).

Parameter	Baseline	Distribution[a]	Lower	Midpoint	Upper
Sedimentation tank solids residence time [d]	45	U	30	-	60
Sedimentation tank maximum COD decay [% of retained]	70%	T	60%	70%	80%
Sedimentation tank MCF decay [% of degraded COD]	80%	T	80%	80%	100%
Sedimentation tank maximum N degradation [% of N removal]	80%	T	70%	80%	90%
Sedimentation tank N_2O emission factor [% of degraded N]	0%	U	0%	-	0.1%
Sedimentation tank length to width ratio	3.3	U	3	-	3.5
Sedimentation tank average width to height ratio	3.6	U	3.3	-	3.8
Sedimentation tank concrete thickness [m]	0.3	U	0.15	-	0.45
Sedimentation tank roof slope [degree]	20	U	10	-	30
Sedimentation tank roof mass [$kg \cdot m^{-2}$]	2.64	U	2.26	-	3.58

[a] U, T, and N represent uniform, triangular, and normal distribution, respectively. For normal distribution, the lower value is μ and the upper value is sigma.

[a] When two values are presented, the first value is for the existing plant and the second value is for the alternative plant.

Table S34. Parameter values, ranges, and distributions unique to Alternative B (total=25).

Parameter	Baseline	Distribution[a]	Lower	Midpoint	Upper
Biogas transfer loss [%]	10%	U	0%	-	20%
Population potentially served by alternative sludge treatment plant	50000	T	45000	50000	55000
Capital cost of alternative plant [USD]	337140	T	303426	337140	370854
Anaerobic baffled reactor hydraulic retention time [d]	3	U	1	-	5
Anaerobic baffled reactor COD removal [% degraded]	93%	U	83%	-	99%
Anaerobic baffled reactor N removal [% of N removed]	8%	U	0%	-	15%
Anaerobic baffled reactor maximum N degradation [% of N removal]	80%	T	70%	80%	90%
Anaerobic baffled reactor N_2O emission factor [% of degraded N]	0%	U	0%	-	0.1%
Anaerobic baffled reactor height [m]	2.5	U	2	-	3
Anaerobic baffled reactor additional concrete [%]	0.25	U	0.2	-	0.3
Anaerobic baffled reactor concrete thickness [m]	0.3	U	0.15	-	0.45
Biogas energy [$kJ \cdot mol\ CH_4^{-1}$]	803	T	802	803	870
Liquid treatment bed hydraulic retention time [d]	3	U	1	-	5
Liquid treatment bed maximum COD removal [% degraded]	70%	T	60%	70%	80%
Liquid treatment bed MCF decay [% of degraded COD]	80%	T	80%	80%	100%
Liquid treatment bed maximum N degradation [% of N removal]	80%	T	70%	80%	90%
Liquid treatment bed N_2O emission factor [% of degraded N]	0%	U	0%	-	0.1%
Liquid treatment bed height [m]	1.5	U	1.2	-	1.8
Liquid treatment bed concrete thickness [m]	0.3	U	0.15	-	0.45
Liquid petroleum gas energy [$MJ \cdot kg^{-1}$]	50	U	49.5	-	50.4
Biogas ecosystem quality CF [$point \cdot kg^{-1}$]	-1.79E-02	T	-1.85E-02	-1.79E-02	-1.72E-02
Biogas human health CF [$point \cdot kg^{-1}$]	-2.70E-02	T	-2.80E-02	-2.70E-02	-2.59E-02
Biogas resources CF [$point \cdot kg^{-1}$]	-1.70E-01	T	-1.71E-01	-1.70E-01	-1.68E-01
Unskilled staff of alternative plant [people]	5	U	0	-	10
Unskilled staff salary [million $UGX \cdot cap^{-1} \cdot month^{-1}$]	750000	U	500,000	-	1
Liquid petroleum gas price [$UGX \cdot kg^{-1}$]	6500	U	6077	-	6667

[a] U, T, and N represent uniform, triangular, and normal distribution, respectively. For normal distribution, the lower value is μ and the upper value is sigma.

[a] When two values are presented, the first value is for the existing plant and the second value is for the alternative plant.

Table S35. Parameter values, ranges, and distributions unique to Alternative C (total=23).

Parameter	Baseline	Distribution[a]	Lower	Midpoint	Upper
UDDT toilet paper [kg·cap^{-1}·h^{-1}]	2.82E-04	U	2.49E-04	-	3.42E-04
UDDT flushing water usage [kg·cap^{-1}·h^{-1}]	0.42	T	0.17	0.42	1.04
UDDT cleansing water usage [kg·cap^{-1}·h^{-1}]	0.042	U	0.015	-	0.13
UDDT desiccant volume [m^{-3}·cap^{-1}·h^{-1}]	8.33E-06	T	8.33E-06	8.33E-06	2.08E-05
UDDT maximum COD removal [% of input]	70%	T	60%	70%	80%
UDDT maximum N degradation [% of input]	80%	T	70%	80%	90%
UDDT MCF aquatic [% of degraded COD]	11%	T	0.4%	11%	27%
UDDT N_2O aquatic emission factor [% of degraded N]	0.5%	T	0.1%	0.5%	7.5%
UDDT desiccant density [kg·m^{-3}]	760	T	663	760	977
UDDT desiccant Mg content [%]	2.24%	T	0.80%	2.24%	5.62%
UDDT desiccant Ca content [%]	30.34%	T	7.42%	30.34%	37.16%
UDDT container collection period [d]	3.5	T	1	3.5	9
UDDT N volatilization [% of total]	5%	U	0%	-	7%
UDDT struvite pK_{sp}	7.57	U	7.3	-	8.1
UDDT precipitate sludge [% of precipitate that settles and can be removed]	75%	U	50%	-	100%
UDDT desired pathogen inactivation [log unit]	2	U	1	-	4
UDDT stored urine pH [pH unit]	9	U	8.9	-	9.1
UDDT MCF decay [% of degraded COD]	10%	T	5%	10%	15%
UDDT N_2O emission factor [% of degraded N]	0%	U	0%	-	0.1%
UDDT minimum feces moisture content after extended storage [%]	10%	U	7%	-	13%
UDDT feces moisture content exponential decay rate constant [d^{-1}]	0.01	U	0.009	-	0.011
UDDT capital cost [USD·toilet^{-1}]	553	U	476	-	630
UDDT annual operating cost [% of capital cost]	10%	U	5%	-	10%
Handcart collection cost [USD·cap^{-1}·d^{-1}]	0.01	U	0.004	-	0.015
Truck transport cost [UGX·m^{-3}]	23000	U	17000	-	30000

[a] U, T, and N represent uniform, triangular, and normal distribution, respectively. For normal distribution, the lower value is μ and the upper value is sigma.

[a] When two values are presented, the first value is for the existing plant and the second value is for the alternative plant.

References

(1) WESP. Country Classification: Data Sources, Country Classifications and Aggregation Methodology. World Economic Situation and Prospects 2014. https://www.un.org/en/development/desa/policy/wesp/wesp_current/2014wesp_country_classification.pdf.

(2) Yoon, S.; Naderpajouh, N.; Hastak, M. Decision Model to Integrate Community Preferences and Nudges into the Selection of Alternatives in Infrastructure Development. *J. Clean. Prod.* **2019**, *228*, 1413–1424. https://doi.org/10.1016/j.jclepro.2019.04.243.

(3) Katukiza, A. Y.; Ronteltap, M.; Oleja, A.; Niwagaba, C. B.; Kansiime, F.; Lens, P. N. L. Selection of Sustainable Sanitation Technologies for Urban Slums — A Case of Bwaise III in Kampala, Uganda. *Sci. Total Environ.* **2010**, *409* (1), 52–62. https://doi.org/10.1016/j.scitotenv.2010.09.032.

(4) Awad, H.; Gar Alalm, M.; El-Etriby, H. Kh. Environmental and Cost Life Cycle Assessment of Different Alternatives for Improvement of Wastewater Treatment Plants in Developing Countries. *Sci. Total Environ.* **2019**, *660*, 57–68. https://doi.org/10.1016/j.scitotenv.2018.12.386.

(5) Kalbar, P. P.; Karmakar, S.; Asolekar, S. R. Life Cycle-Based Decision Support Tool for Selection of Wastewater Treatment Alternatives. *J. Clean. Prod.* **2016**, *117*, 64–72. https://doi.org/10.1016/j.jclepro.2016.01.036.

(6) Kalbar, P. P.; Karmakar, S.; Asolekar, S. R. Assessment of Wastewater Treatment Technologies: Life Cycle Approach. *Water Environ. J.* **2013**, *27* (2), 261–268. https://doi.org/10.1111/wej.12006.

(7) Khattiyavong, C.; Lee, H. S. Performance Simulation and Assessment of an Appropriate Wastewater Treatment Technology in a Densely Populated Growing City in a Developing Country: A Case Study in Vientiane, Laos. *Water* **2019**, *11* (5), 1012. https://doi.org/10.3390/w11051012.

(8) Lizot, M.; Goffi, A. S.; Thesari, S. S.; Trojan, F.; Afonso, P. S. L. P.; Ferreira, P. F. V. Multi-Criteria Methodology for Selection of Wastewater Treatment Systems with Economic, Social, Technical and Environmental Aspects. *Environ. Dev. Sustain.* **2020**. https://doi.org/10.1007/s10668-020-00906-8.

(9) Kalbar, P. P.; Karmakar, S.; Asolekar, S. R. Selection of an Appropriate Wastewater Treatment Technology: A Scenario-Based Multiple-Attribute Decision-Making Approach. *J. Environ. Manage.* **2012**, *113*, 158–169. https://doi.org/10.1016/j.jenvman.2012.08.025.

(10) Kamble, S. J.; Singh, A.; Kharat, M. G. A Hybrid Life Cycle Assessment Based Fuzzy Multi-Criteria Decision Making Approach for Evaluation and Selection of an Appropriate Municipal Wastewater Treatment Technology. *Euro-Mediterr. J. Environ. Integr.* **2017**, *2* (1), 9. https://doi.org/10.1007/s41207-017-0019-8.

(11) Kalbar, P. P.; Karmakar, S.; Asolekar, S. R. Technology Assessment for Wastewater Treatment Using Multiple-Attribute Decision-Making. *Technol. Soc.* **2012**, *34* (4), 295–302. https://doi.org/10.1016/j.techsoc.2012.10.001.

(12) Padrón-Páez, J. I.; Almaraz, S. D.-L.; Román-Martínez, A. Sustainable Wastewater Treatment Plants Design through Multiobjective Optimization. *Comput. Chem. Eng.* **2020**, *140*, 106850. https://doi.org/10.1016/j.compchemeng.2020.106850.

(13) Zhang, F.; Ju, Y.; Dong, P.; Wang, A.; Santibanez Gonzalez, E. D. R. Multi-Period Evaluation and Selection of Rural Wastewater Treatment Technologies: A Case Study. *Environ. Sci. Pollut. Res.* **2020**, *27* (36), 45897–45910. https://doi.org/10.1007/s11356-020-10307-z.

(14) Promentilla, M. A. B.; Janairo, J. I. B.; Yu, D. E. C.; Pausta, C. M. J.; Beltran, A. B.; Huelgas-Orbecido, A. P.; Tapia, J. F. D.; Aviso, K. B.; Tan, R. R. A Stochastic Fuzzy Multi-Criteria Decision-Making Model for Optimal Selection of Clean Technologies. *J. Clean. Prod.* **2018**, *183*, 1289–1299. https://doi.org/10.1016/j.jclepro.2018.02.183.

(15) Singhirunnusorn, W.; Stenstrom, M. K. Appropriate Wastewater Treatment Systems for Developing Countries: Criteria and Indictor Assessment in Thailand. *Water Sci. Technol.* **2009**, *59* (9), 1873–1884. https://doi.org/10.2166/wst.2009.215.
(16) Vidal, B.; Hedström, A.; Barraud, S.; Kärrman, E.; Herrmann, I. Assessing the Sustainability of On-Site Sanitation Systems Using Multi-Criteria Analysis. *Environ. Sci. Water Res. Technol.* **2019**, *5* (9), 1599–1615. https://doi.org/10.1039/C9EW00425D.
(17) Livia, S.; María, M.-S.; Marco, B.; Marco, R. Assessment of Wastewater Reuse Potential for Irrigation in Rural Semi-Arid Areas: The Case Study of Punitaqui, Chile. *Clean Technol. Environ. Policy* **2020**, *22* (6), 1325–1338. https://doi.org/10.1007/s10098-020-01874-3.
(18) Sadr, S. M. K.; Saroj, D. P.; Mierzwa, J. C.; McGrane, S. J.; Skouteris, G.; Farmani, R.; Kazos, X.; Aumeier, B.; Kouchaki, S.; Ouki, S. K. A Multi Expert Decision Support Tool for the Evaluation of Advanced Wastewater Treatment Trains: A Novel Approach to Improve Urban Sustainability. *Environ. Sci. Policy* **2018**, *90*, 1–10. https://doi.org/10.1016/j.envsci.2018.09.006.
(19) Salisbury, F.; Brouckaert, C.; Still, D.; Buckley, C. Multiple Criteria Decision Analysis for Sanitation Selection in South African Municipalities. *Water SA* **2018**, *44* (3), 448–458. https://doi.org/10.4314/wsa.v44i3.12.
(20) Anaokar, G.; Khambete, A.; Christian, R. Evaluation of a Performance Index for Municipal Wastewater Treatment Plants Using MCDM – TOPSIS. *IJTech - Int. J. Technol.* **2018**, *9* (4).
(21) Mucha, Z.; Generowicz, A.; Wójcik, W.; Jóźwiakowski, K.; Baran, S. Application of Multi-Criterial Analysis to Evaluate the Method of Utilization of Sludge from Small Wastewater Treatment Plants with Sustainable Development of Rural Areas. *Environ. Prot. Eng.* **2016**, *Vol. 42* (nr 4). https://doi.org/10.5277/epe160408.
(22) McConville, J. R.; Kvarnström, E.; Nordin, A. C.; Jönsson, H.; Niwagaba, C. B. Structured Approach for Comparison of Treatment Options for Nutrient-Recovery From Fecal Sludge. *Front. Environ. Sci.* **2020**, *8*. https://doi.org/10.3389/fenvs.2020.00036.
(23) Perez, A.; Mena, M.; Oddershede, A. Wastewater Treatment System Selection Using the Analytical Hierarchy Process; 2010; pp 130–137.
(24) Seleman, A.; Bhat, M. G. Multi-Criteria Assessment of Sanitation Technologies in Rural Tanzania: Implications for Program Implementation, Health and Socio-Economic Improvements. *Technol. Soc.* **2016**, *46*, 70–79. https://doi.org/10.1016/j.techsoc.2016.04.003.
(25) Willetts, J.; Paddon, M.; Nam, N.; Trung, N.; Carrard, N. Sustainability Assessment of Sanitation Options in Vietnam: Planning with the Future in Mind. *J. Water Sanit. Hyg. Dev.* **2013**, 262–268. https://doi.org/10.2166/washdev.2013.045.
(26) Gao, H.; Zhou, C.; Li, F.; Han, B.; Li, X. Economic and Environmental Analysis of Five Chinese Rural Toilet Technologies Based on the Economic Input–Output Life Cycle Assessment. *J. Clean. Prod.* **2017**, *163*, S379–S391. https://doi.org/10.1016/j.jclepro.2015.12.089.
(27) Singh, S.; Mohan, R. R.; Rathi, S.; Raju, N. J. Technology Options for Faecal Sludge Management in Developing Countries: Benefits and Revenue from Reuse. *Environ. Technol. Innov.* **2017**, *7*, 203–218. https://doi.org/10.1016/j.eti.2017.02.004.
(28) Hashemi, S.; Boudaghpour, S. Economic Analysis and Probability of Benefit of Implementing Onsite Septic Tank and Resource-Oriented Sanitation Systems in Seoul, South Korea. *Environ. Technol. Innov.* **2020**, *18*, 100762. https://doi.org/10.1016/j.eti.2020.100762.
(29) Kerstens, S. M.; Leusbrock, I.; Zeeman, G. Feasibility Analysis of Wastewater and Solid Waste Systems for Application in Indonesia. *Sci. Total Environ.* **2015**, *530–531*, 53–65. https://doi.org/10.1016/j.scitotenv.2015.05.077.
(30) Retamal, M.; Willetts, J.; Mitchell, C.; Carrard, N. Modelling Costs for Water and Sanitation Infrastructure: Comparing Sanitation Options for Can Tho, Vietnam. **2011**.

(31) Spuhler, D.; Scheidegger, A.; Maurer, M. Generation of Sanitation System Options for Urban Planning Considering Novel Technologies. *Water Res.* **2018**, *145*, 259–278. https://doi.org/10.1016/j.watres.2018.08.021.
(32) Spuhler, D.; Germann, V.; Kassa, K.; Ketema, A. A.; Sherpa, A. M.; Sherpa, M. G.; Maurer, M.; Lüthi, C.; Langergraber, G. Developing Sanitation Planning Options: A Tool for Systematic Consideration of Novel Technologies and Systems. *J. Environ. Manage.* **2020**, *271*, 111004. https://doi.org/10.1016/j.jenvman.2020.111004.
(33) Simiyu, S. Preference for and Characteristics of an Appropriate Sanitation Technology for the Slums of Kisumu, Kenya. *Int. J. Urban Sustain. Dev.* **2017**, *9* (3), 300–312. https://doi.org/10.1080/19463138.2017.1325366.
(34) Triantafyllidis, C. P.; Koppelaar, R. H. E. M.; Wang, X.; van Dam, K. H.; Shah, N. An Integrated Optimisation Platform for Sustainable Resource and Infrastructure Planning. *Environ. Model. Softw.* **2018**, *101*, 146–168. https://doi.org/10.1016/j.envsoft.2017.11.034.
(35) Malekpour, S.; Langeveld, J.; Letema, S.; Clemens, F. Judgment under Uncertainty; a Probabilistic Evaluation Framework for Decision-Making about Sanitation Systems in Low-Income Countries. *J. Environ. Manage.* **2013**, *118C*, 106–114. https://doi.org/10.1016/j.jenvman.2013.01.007.
(36) Abdel Wahaab, R.; Mahmoud, M.; van Lier, J. B. Toward Achieving Sustainable Management of Municipal Wastewater Sludge in Egypt: The Current Status and Future Prospective. *Renew. Sustain. Energy Rev.* **2020**, *127* (C).
(37) Schwab, K.; Xavier Sala-i-Martin; Richard Samans. *The Global Competitiveness Report 2017-2018*; World Economic Forum: Geneva, 2017; p 393.
(38) Geneva: World Health Organization (WHO) and the United Nations Children's Fund (UNICEF). Progress on Household Drinking Water, Sanitation and Hygiene 2000–2020: Five Years into the SDGs, 2021.
(39) International Labour Organization. *ILOSTAT*. https://ilostat.ilo.org/data/ (accessed 2020-08-15).
(40) The World Bank. *World Bank Open Data*. https://data.worldbank.org/ (accessed 2020-08-15).
(41) World Resources Institute. *Beta Aqueduct Country Rankings*. https://www.wri.org/applications/aqueduct/country-rankings/ (accessed 2020-08-15).
(42) Trimmer, J. T.; Guest, J. S. Recirculation of Human-Derived Nutrients from Cities to Agriculture across Six Continents. *Nat. Sustain.* **2018**, *1* (8), 427–435. https://doi.org/10.1038/s41893-018-0118-9.
(43) Trimmer, J. T.; Lohman, H. A. C.; Byrne, D. M.; Houser, S. A.; Jjuuko, F.; Katende, D.; Banadda, N.; Zerai, A.; Miller, D. C.; Guest, J. S. Navigating Multidimensional Social–Ecological System Trade-Offs across Sanitation Alternatives in an Urban Informal Settlement. *Environ. Sci. Technol.* **2020**, *54* (19), 12641–12653. https://doi.org/10.1021/acs.est.0c03296.
(44) Pérez, A.; Mena, M. Wastewater Treatment System Selection Using the Analytical Hiearchy Process. In *Extended Abstracts*; 2010.
(45) Tilley, E.; Ulrich, L.; Lüthi, C.; Reymond, P.; Zurbrügg, C. Compendium of Sanitation Systems and Technologies. Swiss Federal Institute of Aquatic Science and Technology (Eawag) 2014. https://www.iwa-network.org/wp-content/uploads/2016/06/Compendium-Sanitation-Systems-and-Technologies.pdf.
(46) Jaibiba, P.; Naga Vignesh, S.; Hariharan, S. Chapter 10 - Working Principle of Typical Bioreactors. In *Bioreactors*; Singh, L., Yousuf, A., Mahapatra, D. M., Eds.; Elsevier, 2020; pp 145–173. https://doi.org/10.1016/B978-0-12-821264-6.00010-3.
(47) Huijbregts, M. A. J.; Steinmann, Z. J. N.; Elshout, P. M. F.; Stam, G.; Verones, F.; Vieira, M.; Zijp, M.; Hollander, A.; van Zelm, R. ReCiPe2016: A Harmonised Life Cycle Impact

Assessment Method at Midpoint and Endpoint Level. *Int. J. Life Cycle Assess.* **2017**, *22* (2), 138–147. https://doi.org/10.1007/s11367-016-1246-y.
(48) Maj, H.; Zjn, S.; Pmf, E.; G, S.; F, V.; Mdm, V.; A, H.; M, Z.; R, van Z. *ReCiPe 2016 : A Harmonized Life Cycle Impact Assessment Method at Midpoint and Endpoint Level Report I: Characterization*; Report; Rijksinstituut voor Volksgezondheid en Milieu RIVM, 2016. https://rivm.openrepository.com/handle/10029/620793 (accessed 2021-08-23).
(49) *Ecoinvent*; 2021.
(50) Quantitative Sustainable Design Group. *BW2QSD*. https://github.com/QSD-Group/BW2QSD (accessed 2021-08-15).
(51) Haasad, A. *EcoInventDownLoader*.
(52) Mutel, C. Brightway: An Open Source Framework for Life Cycle Assessment. *J. Open Source Softw.* **2017**, *2* (12), 236. https://doi.org/10.21105/joss.00236.
(53) Quantitative Sustainable Design Group. *EXPOsan*. https://github.com/QSD-Group/EXPOsan (accessed 2021-08-15).
(54) Quantitative Sustainable Design Group. *QSDsan*. https://github.com/QSD-Group/QSDsan (accessed 2021-08-15).
